# A climate action intervention increases four psychological motivators, as well as climate advocacy, activism and civic participation

**DOI:** 10.1098/rsos.261346

**Published:** 2026-10-21

**Authors:** Anna Castiglione, Cameron Brick, Anna Sach, Gianluca Esposito, Andrea Bizzego

**Affiliations:** ^1^ Department of Psychology and Cognitive Science, University of Trento, Trento, Italy; ^2^ Department of Psychology, University of Amsterdam, Amsterdam, The Netherlands

**Keywords:** climate change, climate crisis, climate action, climate activism, pro-climate behaviour, climate anxiety

## Abstract

Urgent climate action is needed to keep warming below 2°C and protect hundreds of millions from climate collapse. As part of a broader Registered Report series, this study examined whether a longitudinal intervention designed to strengthen psychological correlates of pro-environmental behaviour could motivate *n* = 31 university students in The Netherlands to take climate action. The intervention was delivered over six weeks through interactive lessons and activities informed by psychological theories and interventions. The goal was to increase pro-climate behaviours by targeting 12 psychological factors. Participants completed surveys assessing these correlates, as well as ecological momentary assessments of individual and collective pro-climate behaviours before and after the intervention, and after three months (14 days each). A control group was included (*n* = 27). Results showed that 4 of the 12 psychological correlates increased significantly: emotional engagement, collective efficacy, the bottom-up theory of change (the belief that change can start from grassroots action and spread systemically) and climate appraisal. Three (except appraisal) remained elevated at the three-month follow-up. Civic engagement, advocacy and activism increased after the intervention in the experimental group relative to control group. The increase in activist behaviour was predicted by the development of a bottom-up theory of change.

## 1. Introduction

Human-driven greenhouse gas emissions have long been known to threaten human well-being, and the solutions are clear: phasing out fossil fuels for energy and transportation, regulating polluting industries and agriculture, and shifting consumption patterns [1,2]. Yet action is lagging, increasing the risk of dangerous tipping points. Effective mitigation requires both government regulations and individual behavioural change, particularly collective engagement to pressure institutions to regulate high-emission industrial practices and consumption [3,4]. Despite the urgency, participation remains minimal, where it is estimated that fewer than 0.01% of people are engaged in grassroots advocacy or activism [5–7]. While some people remain unaware that a climate crisis exists [8], many face material constraints to engagement, such as unmet basic needs, limited time or resources, or lack of information about available climate initiatives [9,10]. Most, however, encounter psychological barriers, including perceiving climate change as temporally or spatially distant [11], viewing power structures as inaccessible [12], doubting the impact of individual action in the face of a global and complex problem [13], or experiencing fear-induced paralysis [14].

While gaps in awareness and material constraints remain important, highlighting the need to strengthen climate education [15,16] and make climate action initiatives more inclusive, accessible and intersectional with other social struggles [17,18], environmental psychology has increasingly focused on the psychological factors that are associated with, and believed to drive, engagement. These factors include, for example, emotional engagement, efficacy beliefs, environmental identity and cognitive alternatives [19,20]. Yet, existing studies in this field have common limitations [13]: they often rely on cross-sectional designs that prevent causal conclusions on the action-triggering role of these factors, and on biased self-reports of past behaviours or hypothetical future intentions, rather than current actions [21,22]. More longitudinal studies employing high-frequency, real-time and ecologically valid measures of behaviour would help identify which psychological factors causally drive behaviour. Although some have emerged in recent years (e.g. [13,23,24]), such studies remain relatively rare, especially in relation to collective climate action.

As part of this programmatic Registered Report series, a previous study tested whether a six-week intervention increased key psychological factors associated with pro-environmental behaviour and, as a result, collective and individual climate action [25]. The intervention increased 10 out of 12 psychological factors, among others, emotional engagement, self-efficacy, collective efficacy and bottom-up theory of change. Additionally, it temporarily increased climate *advocacy* (including contacting decision makers, joining climate advocacy organizations and speaking up during electoral events) and *sustainable food consumption* and supported *climate education behaviours* up to the follow-up (including informing friends about climate actions and posting about climate issues/actions on social media). The effects on behaviour were predicted by combinations of emotional engagement, self-efficacy, collective efficacy, bottom-up theory of change, collective identity and cognitive alternatives.

While successful in increasing action, the previous intervention had several limitations. First, over the long term, it increased only lower-effort behaviours (e.g. climate education) and not higher-cost actions such as advocacy, activism or civic participation. For example, advocacy increased immediately following the intervention but not at follow-up. Participants reported that key barriers to sustaining behaviour after the intervention period included a lack of connection with real-world activists and insufficient practical information about activist events they could join. Second, the study was underpowered owing to low participant compliance with the daily behavioural tracking (ecological momentary assessment) and extensive data exclusions. These exclusions were owing to the use of open-ended rather than forced-choice items in the daily surveys, which yielded many responses that were not directly relevant to climate action. Consistent with prior evidence, in the absence of clear guidance, people often misjudge which behaviours meaningfully reduce greenhouse gas emissions (e.g. overemphasizing recycling; [26,27]) and what constitutes advocacy or activism [10]. Third, participants wished to receive more information about past successes of collective climate action and to spend more time engaging in real-world climate activities during the intervention. Climate education interventions that incorporate activities with tangible real-world impact, such as participation in existing community initiatives, tend to have strong motivational and mobilizing effects [28,29]. Finally, the results may not generalize to young adults outside the Italian context in which the study was conducted. Overall, these limitations highlight the need for further research to determine which psychological factors best support sustained student engagement in climate action and how they can be effectively incorporated into climate education, as well as to test these approaches across diverse cultural contexts.

In studying the psychological motivators of collective climate action, a particularly key population is young adults, for at least three reasons. First, young adults like university students have historically played an important role in driving cultural change, raising awareness of civil rights, opposing wars and advancing climate activism, and could mobilize similarly for climate mitigation if they perceived the urgency of the crisis [30,31]. Second, they are at a formative stage of making career choices, which could be oriented towards climate solutions if guided by appropriate interventions [32]. Third, they act as key knowledge diffusers, engaging not only with academic communities but also with family, peers outside academia and wider cultural networks such as sports and arts communities, thereby amplifying the reach of collective climate engagement [33]. In addition to these theoretical motivations, university students also represent a practically feasible population for implementing longitudinal interventions involving repeated in-person sessions and behavioural tracking.

### 1.1. This study

In this study, we built on [25] to optimize a longitudinal intervention designed to activate students’ engagement in climate action, using participant feedback and prior results to guide improvements. We also extended the investigation to a new cultural context by focusing on university students in The Netherlands. Compared to Italy, where [25] took place, The Netherlands differs in relevant aspects such as a long history of socially normalized environmental activism, which contributed to a developed cycling culture and extensive public transportation infrastructure [34] while remaining sufficiently comparable as a European university context to allow a meaningful extension of the previous Italian study. Additionally, conducting the study in The Netherlands was practically feasible given that some authors were affiliated with Dutch academic institutions. We included new activities such as connecting participants with real-world activists and providing information about ongoing local activist events via a dedicated social media channel. Additionally, we redesigned the behavioural tracking procedure, namely ecological momentary assessment (EMA), used in [25], adopting a forced-choice format to guide participants in selecting relevant behaviours, in four categories for collective climate actions (civic participation, advocacy, activism and education) and four categories for individual actions (transportation, food, energy and finance). The updated intervention also emphasized success stories of collective climate activism and included more time dedicated to designing real-world campaigns. All anonymized data and codes are available at the Open Science Framework: https://osf.io/9kve7/files/osfstorage?view_only=02aafa977bd64a2aac6158d33b4e87f3.

## 2. Methods

The present study is part of an incremental, programmatic series of Registered Reports comprising multiple studies, of which this manuscript reports the second. Following this format, an initial registered study was conducted with university students in Italy. Based on the results of that first study, the intervention was subsequently optimized and implemented in the present study with university students in The Netherlands. In this study, we used a pre–post design with a control group who completed the same questionnaires as the experimental group but did not undergo the same intervention.

### 2.1. Recruitment

The final sample was *n* = 58, with *n* = 31 experimental and *n* = 27 control participants. Initially, *n* = 130 participants were recruited, with *n* = 63 experimental participants and *n* = 67 control participants, via flyers posted at the University of Amsterdam, class presentations and email announcements. This initial sample size resulted from our recruitment efforts, which originally aimed for *n* = 102 (the most conservative estimate in our power analysis), evaluating each of our three hypotheses separately (see §2.2, ‘Power analysis’) and considering a 40% dropout for the experimental participants and a 35% dropout for the control participants, based on prior literature [25].

Participants in the experimental group were invited to attend six 3-hour climate education sessions and complete online questionnaires. The control group was recruited to complete the same pre–post questionnaires as the experimental group without attending the in-person sessions. The control group was a mixed active/passive group, that is 12 control participants (45%) were students enrolled in in-person courses related to the environment/climate happening at the same time as the intervention, while 15 control participants (55%) did not attend an alternative in-person educational programme. Owing to difficulties in recruiting participants exclusively from environmental courses, a mixed control condition was adopted. Initially, we automatically directed to the control group any student who, during recruitment, declared being enrolled in an environmental course taking place during the study period. However, owing to insufficient recruitment, we subsequently opened control-group enrolment to participants not attending environment-related courses.

All participants were invited to fill out a screening questionnaire online. Students could participate if they fulfilled the screening criteria: (i) being 18–35 years old, (ii) being proficient in English, (iii) believing in climate change, and (iv) not being civically engaged on the climate topic, i.e. not having participated in more than two climate-related civic initiatives in the past three months). Those who passed the screening were automatically enrolled in a raffle system in which participants were eligible to win either one of three €150 prizes or one of seven €50 prizes, depending on study condition. The larger prizes were allocated to the experimental condition to reflect the greater participant workload (see the supplementary material). Participants in the experimental group who successfully completed all the intervention stages also obtained a certificate of participation.

The final 58 participants were 71% female, 26% male and 3% non-binary. Their age was *M* (s.d.) = 22.9 (3.5) years, range 18–34. Regarding political orientation, 76% were affiliated to the left, 9% to the centre, 5% to the right, 4% were apolitical and 5% undeclared. The sample encompassed relatively diverse countries of origin, with 78% being from a European country, 14% from Asia, 8% from North America and 2% from Africa.

### 2.2. Power analysis

*A priori* power analyses were pre-registered at stage 1. In the H1 analysis, we tested whether each psychological factor increased from before to after the intervention, across conditions, via a general linear model (GLM). The power analysis focused on this condition × time interaction effect. We used the R package *pwr* [35] with *f*^2^= 0.09 (*d* = 0.60), alpha = 0.05, power = 0.85 and numerator df 1 (the condition × time interaction). We chose *f*^2^ = 0.09 as the smallest effect size of interest [36] for a manipulation-induced psychological change that we expected to be the minimum needed to lead to a behavioural change. Similar effect sizes were found in manipulation studies inducing a change in one of the psychological factors tested here (e.g. [13,24,37,38]). A smaller psychological effect may not trigger subsequent behavioural change and, therefore, would be less interesting for us. This power analysis yielded a total *n* = 101 (about 51 participants per condition).

In the H2 analysis, we tested whether behaviour increased from baseline to final for the experimental versus the control group via a linear mixed effects model. Again, the power analysis focused on this condition × time interaction effect. We used PANGEA (v.0.2) to perform a power analysis (2 × 3 mixed, within × between method), with effect size *d* = 0.25, power = 0.85 and replicates = 14. The effect size was based on a recent meta-analysis on behavioural interventions that promote climate action, which found effect sizes ranging *d*  =  0.25–0.27 [39]. This power analysis yielded *n* = 100 (50 participants per condition).

In the H3 analysis, we tested whether any change in behaviour was predicted by a change in any of the target psychological factors via a multiple linear regression model; we used the R package *pwr*, with *f*^2^ = 0.15 (*d* = 0.78), alpha = 0.05, power = 0.85 and numerator df = 5 (assuming only some of the psychological factors would enter this analytical phase). We chose *f*^2^ = 0.15 aggregating across the literature on the psychological correlates and pro-environmental behaviours (e.g. [19,20,40–42]). This power analysis yielded a total *n* = 102 (51 participants per condition).

### 2.3. Exclusion criteria

Participants were excluded sequentially according to the following criteria. First, experimental participants who attended fewer than three out of six intervention meetings were excluded (*n* = 29). Among the remaining participants (experimental and control), those who failed to complete either the baseline psychological questionnaire or the final psychological questionnaire were excluded (*n* = 12). Then, those who completed fewer than five daily EMA surveys out of 14 during the baseline EMA period were excluded (*n* = 31). Finally, participants who completed fewer than five daily EMA surveys during either the final (*n* = 7) or follow-up EMA tracking stage (*n* = 23) were only excluded from the EMA analyses corresponding to those stages.

### 2.4. Design

#### 2.4.1. Timeline

The study lasted 12 weeks, including the intervention (six weeks) and the behavioural tracking periods (two weeks before the intervention, two weeks after the intervention and two weeks after three months) (figure 1). First, participants were recruited to be part of either the experimental or control group (random assignment was impossible owing to recruitment challenges). Then, *baseline* psychological and behavioural measures were collected for all participants: the 12 psychological factors and other psychological variables were measured via online questionnaires, and pro-climate behaviour was measured via EMA for two weeks, that is 14 daily surveys tracking same-day behaviours. For the following six weeks, the experimental group attended the intervention. Every week the participants met in person to attend a 3 hour module on a specific topic regarding climate change and some module-validation questionnaires. After the intervention, the *final* psychological and behavioural measures were collected for all participants (questionnaire for the 12 psychological factors and two-week EMA for behaviour). Additionally, participants of the experimental group could participate in a focus group. The same measures (psychological and behavioural measures) were collected again at *follow-up,* three months after the intervention.

**Figure 1.**
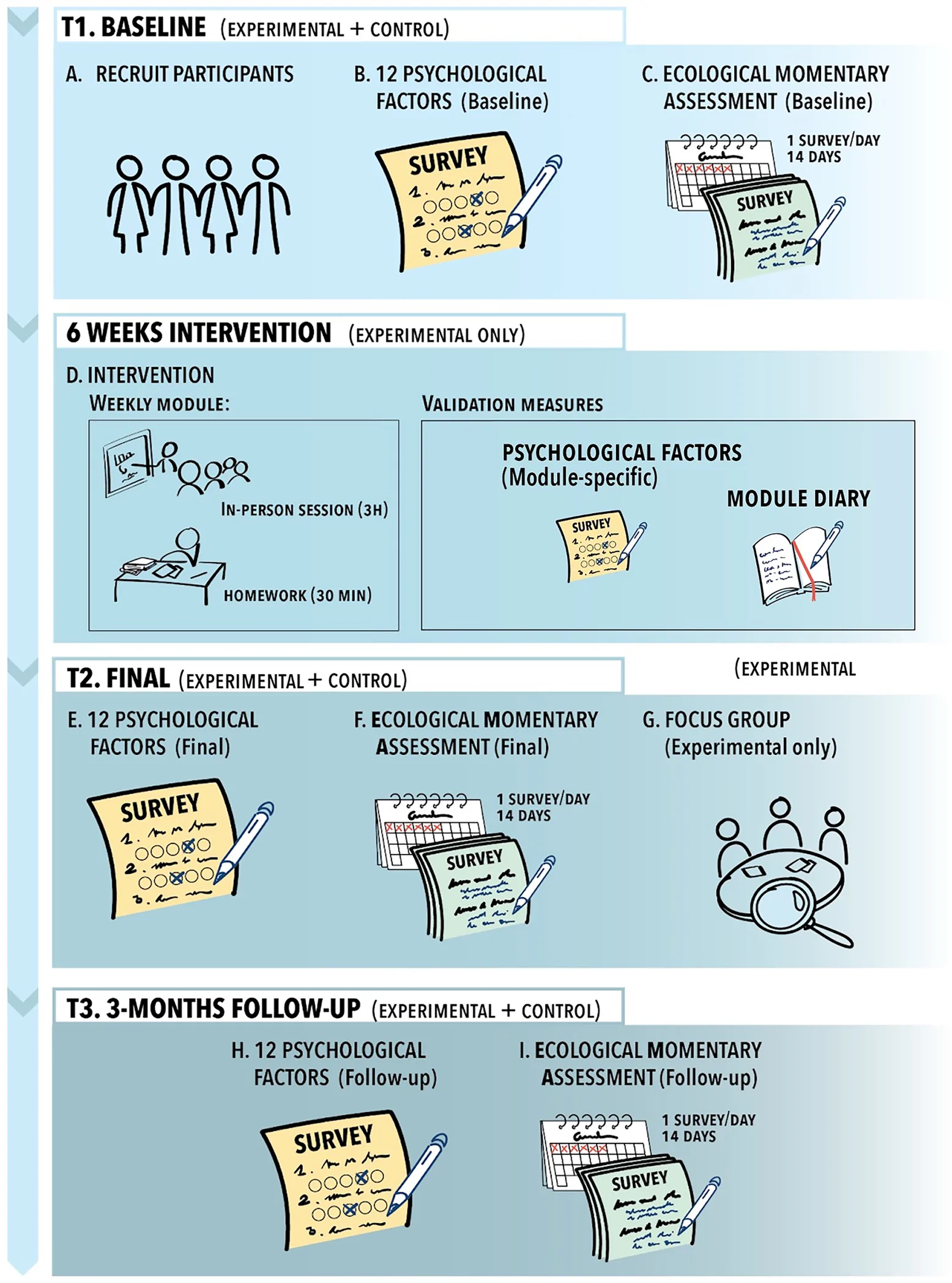
Study design.

#### 2.4.2. Baseline: psychological measures

All participants received an online questionnaire, measuring (i) 12 psychological factors (table 1), (ii) climate anxiety (to monitor people's potential undue distress), (iii) behavioural recall for the last three months, and (iv) demographics (figure 1B). See the supplementary material for the full measures.

##### 2.4.2.1. The 12 psychological factors:

For each factor, participants rated multiple items, adapted from environmental psychology studies, by modifying some language and adding some items to address psychological dynamics and behaviours related to climate change specifically (see the supplementary material for the full questionnaire and references). Definitions and example items are provided for each psychological factor in table 1.

##### 2.4.2.2. Climate anxiety:

This 20-item questionnaire (see the supplementary material) was used to monitor people's potential distress when exposed to material related to the climate crisis (modified from [68]).

##### 2.4.2.3. Behavioural recall:

Participants reported the frequency with which they performed the targeted individual and collective climate behaviours over the previous three months (see the supplementary material).

#### 2.4.3. Baseline: ecological momentary assessment

Participants filled out a daily 10 minute survey on their phone, reporting their pro-climate behaviours during the same day, for two weeks. To maximize compliance, participants were given an additional week before the intervention began (a buffer week) during which they could complete extra EMA surveys if they had missed any in the first two weeks. The daily diaries were implemented in Qualtrics XM [69]. Participants received the survey link every evening at 17.00 and had until midnight to complete it. Before the EMA period started, they read instructions describing the behaviours they were asked to report on and the EMA procedure. We measured two behaviour types: collective (behaviours typically performed with other people and having a collective impact) and individual (behaviours performed alone, with an impact typically limited to that one action). For each behaviour type, we grouped all the relevant climate mitigation behaviours into four categories (see table 2 for some examples). Collective behaviours included the following: (i) civic participation (i.e. participating in existing civic initiatives regarding climate mitigation), (ii) advocacy (formally negotiating with institutions for emission reduction policy), (iii) activism (organizing grassroots pressure on institutions for emission reduction policy), and (iv) education (educating others about climate mitigation). Individual behaviours included reducing one's carbon footprint from (i) food consumption, (ii) transportation, (iii) energy use, and (iv) finances (e.g. which products and services they buy). The behavioural categories assessed were derived from prior literature identifying key leverage points for climate mitigation. Collective behaviours were selected based on literature and applied resources highlighting their role in influencing institutional decision-making and enabling large-scale climate mitigation (e.g. [3,70,71]). Individual behaviours were selected based on their documented contribution to reduce greenhouse gas emissions (e.g. [72,73]).

In the instructions, participants were informed that every evening they would receive a survey link on their phone asking them to (i) report pro-climate behaviours performed on the same day (action section), and (ii) plan pro-climate behaviours for the following day (plan section). We wanted to track planning as a fundamental precursor of behaviour, which we hoped to strengthen via the intervention [55]. In the *plan* section, for each category, participants could select from a drop-down window up to five mitigation behaviours that they planned to do the next day. They could also choose not to select any behaviour or add additional behaviours not listed as open answers. In the *action* section, participants were shown the same action they selected in the previous day *plan* and they selected ‘yes’ if they did it or ‘no’ if they did not. There was also an option to add any unplanned pro-climate behaviours they performed.

**Table 1.** Definitions and example items for the 12 psychological factors.

factor	definition	example item
*emotional engagement*	feeling deeply emotionally aroused by the climate crisis, experiencing emotions such as fear, concern, personal risk and moral outrage [43,44]	‘when thinking about the climate crisis, I feel: fear, guilt, sadness, shame…’ (none at all–very strongly) [45]
*self-efficacy*	the perception of possessing the skills and competence needed to carry out a particular task (i.e. writing a petition, speaking in public and using social media) [41]. The conviction that one's efforts will significantly further the group's objectives [46]	‘to which extent do you feel capable of supporting a climate initiative/campaign/petition by signing it off or donating to the cause?’ from (not at all–very much) [41]
*collective efficacy*	the belief that one's group can enact a desired change, either directly or indirectly [47–50]. The sensation of accomplishment in the group's attainment of goals and in consolidating collective power	‘our pro-climate actions as students will encourage others to do the same.’ (strongly disagree–strongly agree) [48]
*theory of change (bottom-up)*	the extent to which individuals believe that successful climate mitigation actions can start (and progress) from the bottom (i.e. from the grassroots) and spread to the top (e.g. government) [13,51,52]	‘climate mitigation can start locally (e.g. in my city, in my school, in my company) and spread globally’, (completely agree–completely disagree) (modified from [13])
*controllability*	the control someone feels over whether or not they engage in a specific behaviour [53]. How easily someone feels they can engage in pro-climate behaviours (e.g. decreasing one's CO_2_ footprint or joining activist events) versus perceiving high instrumental barriers to engagement (i.e. lack of time, money and resources to engage) [54]	‘how much control do you have over whether you reduce your personal emissions (from diet, transport, energy, and finances)?’ (very little control–a great deal of control) (modified from [54])
*implementation intentions*	the ability to plan the practical execution of one's intentions to act. This entails outlining practical action steps through an ‘if, then’ plan. For instance, planning actions like attending a particular climate event at a designated time on a specific day [55]	‘I have plans on when, where and how I will engage civically to vote, sign petitions or support campaigns in favor of a climate issue’, (completely agree–completely disagree) (modified from [55])
*social norm*	how an individual perceives the opinions of valued others regarding their pro-environmental conduct [56–58]. Feeling inclined (or discouraged) to engage in actions based on the support (or disapproval) received from friends, peers or family [54]	‘most people who are important to me (friends, family, peers) would approve if I engaged collectively to mitigate climate change, such as educating others, supporting civic initiatives, doing advocacy or activism’, (completely agree–completely disagree) (modified from [54])
*environmentalist self*-*identity*	the degree of connection a person experiences with the broader natural environment and their sense of interconnectedness with nature [59]. How strongly an individual identifies as an environmentalist and embraces this identity with positivity [60,61]	‘engaging collectively with other citizens to mitigate the climate crisis is important to my self-concept’, (completely agree–completely disagree) (modified from [61])
*environmentalist collective identity*	experiencing a strong bond with a community of activists [19,62,63]. Feeling a kinship with the group, owing to shared objectives or demographic similarities such as ethnicity, socio-economic status, age, beliefs, gender identity, LGBTQIA+ affiliation or political stance. Nurturing close social connections within the organization [64]	‘I feel strong ties with other environmentalists’, (completely agree–completely disagree) [19]
*trust in institutional politics*	the perception that political institutions keep public opinion and individual citizens’ efforts into account in the decision and policy-making process [65] and that citizens can have a say in these processes [66]	‘people like me don't have any say about what the government does on climate change’, (completely agree–completely disagree) [66]
*appraisal*	the understanding that a problem exists (e.g. that climate change exists, that it is caused by humans and that it is dangerous for us) before deciding to address this problem [63,67]	‘it is a serious problem that increasing global temperatures are leading to more catastrophic extreme weather events’, (completely agree–completely disagree) [67]
*cognitive alternatives*	the ability to imagine cognitive alternatives to the environmental status quo, i.e. how easily people can imagine what that sustainable world would be/look like [20]	‘it is easy to imagine a world where we no longer use fossil fuels’, (completely disagree–completely agree) [20]

**Table 2.** Behaviour types, categories and examples.

behaviour type	behaviour category	examples
collective	civic participation	participating in existing civic initiatives such as signing a petition, donating to a climate campaign and joining a climate march
	advocacy	negotiating formally with politicians and administrators to introduce and apply climate mitigation policy, via non-governmental organizations, formal events such as city councils or by directly contacting them
	activism	organizing grassroots initiatives such as campaigns, petitions, marches, protests and flash mobs, to *put pressure* on politicians and administrators to introduce and apply climate mitigation policy
	education	organizing climate education events/campaigns/programmes or informing others of climate contents via social media
individual	food consumption	reducing/cutting meat and dairy products from one's diet or reducing/cutting the consumption of non-local products
	transportation	opting for clean transports such as bikes, scooters, electric transports, public transports, walking and car-pooling rather than driving internal combustion cars or flying
	energy conservation	limiting appliance use to the strictly necessary, reducing heating/cooling and hot water use, unplugging appliances and switching lights off
	finances	buying only necessary goods, opting for low-carbon services such as home utilities from 100% renewable sources and banking/insurance services with banks/insurances not tied to the fossil fuel industry

#### 2.4.4. Intervention

The experimental group attended a six-week intervention, which was designed to increase pro-climate behaviour by boosting 12 psychological factors associated with pro-environmental behaviour (figure 1). The participants attended six 3-hour meetings in a classroom of the University of Amsterdam. Each meeting addressed one module, regarding a specific aspect of the climate crisis and climate action. In each module, we aimed to boost two to four psychological factors via educational content and group activities. An overview of the module topics, description and example activities with references can be found in the supplementary material. Before each meeting, the participants received a preparatory ‘homework’ email, with some text/video/podcast introducing the module. In the meeting, the participants listened to a 1 hour presentation by a tutor, laying out the core topic of the module. For the rest of the time, they carried out individual and group activities, such as sharing circles on beliefs, emotions, behaviours and experiences related to the climate crisis, discussions of video contents, small research projects or activities to plan for personal and institutional decarbonization (see the supplementary material for a full description of the activities). At the end of the meeting, they answered a few comprehension questions to verify they were paying attention during the module and completed a module-validation questionnaire and a module diary where they reported their impressions of the module materials.

##### 2.4.4.1. Topics of the six modules:

The topics of the six modules were as follows: (i) *Intro to the climate crisis and climate action*, addressing the causes and impacts of the climate crisis and the psychological obstacles as well as incentives to mitigation behaviours; (ii) *Decarbonization*, on the main greenhouse gasses, their anthropogenic sources and how to reduce them; (iii) *Climate economics*, on switching from a linear extractivist economy to a post-growth economy; (iv) *Systemic change*, on how societal change can spread from the local to the global; (v) *Civic engagement for the climate*, on how to create effective bottom-up change; and (vi) *Teamwork for civic engagement*, on effective teamwork for climate action.

##### 2.4.4.2. Factor-boosting activities:

These activities were inspired by previous psychological interventions and theories and were designed to boost the 12 psychological factors of interest. Some activities replicate existing manipulations that were shown to successfully raise a specific factor of interest (e.g. [37] for self-efficacy, [38] for emotional engagement and [74] for social norms). Other activities we created ex novo, and they were either inspired by existing theories (e.g. [75] for emotional engagement, [76] and [41] for self-efficacy and [77] for collective identity) or from the authors’ experiences in climate advocacy. The activities were distributed across the six modules coherently with the core topic of the module. See the supplementary material table S1 for a full list of the activities in the intervention modules.

#### 2.4.5. Final: psychological measures, ecological momentary assessment and focus group

At the end of the intervention, all participants completed a final survey, similar to the baseline survey, including the 12 psychological factors and climate anxiety (but not demographics and behavioural recall questions; figure 1E). All participants completed two weeks of EMA behavioural tracking, with an additional buffer week, as in the baseline stage (figure 1F). The participants from the experimental group were invited to a final focus group session held online, during which they shared their experiences in the intervention (figure 1G).

#### 2.4.6. Follow-up: psychological measures and ecological momentary assessment

Three months after the end of the study, all participants filled out the same psychological questionnaire online, including the 12 psychological factors, climate anxiety and behavioural recall (figure 1H). They also completed two more weeks of EMA behavioural tracking, plus one buffer week (figure 1I).

### 2.5. Measures

#### 2.5.1. Behavioural scores

For both individual and collective tracked behaviours, we calculated three scores per participant for each day of each EMA period (baseline, final and follow-up). The *action score* was the absolute number of actions participants performed that day, summed across all eight categories. This included actions that were not planned the day before. The *plan score* was the number of actions they had planned the day before to be executed that day. If participants did not complete any part of the survey (action or plan), we assigned ‘NA’ to that part of the survey. The *alignment score* was calculated as the difference between the actions actually performed and the actions planned the day before (e.g. three performed minus five planned would be −2). For behavioural recall (the self-reported frequency of individual and collective climate behaviours over the previous three months, measured in the baseline and follow-up questionnaires), we calculated the mean of the 1–10 ratings for individual and collective behaviours, resulting in one composite per behaviour type.

#### 2.5.2. Psychological scores

For each of the 12 psychological factors, we calculated the mean of the 1–10 ratings across that factor's items, resulting in one composite score per factor. McDonald's omega was calculated for reliability.

## 3. Hypotheses

### 3.1. H1: pre–post psychological factors

Some of the 12 psychological factors will increase from baseline to final significantly more for the experimental group versus control.

### 3.2. H2: pre–post behaviour

Individual and collective climate action will increase owing to the intervention (i.e. significantly more for the experimental group versus control): tracked behaviour via EMA will increase from baseline to final and from baseline to follow-up (H2a); planning and the alignment between planning and action will increase from baseline to final and from baseline to follow-up (H2b); and behavioural recall will increase from baseline to follow-up (H2c).

### 3.3. H3: behaviour change associated with psychological change

If any change in individual and/or collective climate action occurs from baseline to final or from baseline to follow-up across all participants, it will be associated with the change in the 12 psychological factors increasing from baseline to final (H3a) and similarly for planning and the alignment between action and planning (H3b).

## 4. Analytic plan

### 4.1. Analyses

#### 4.1.1. Pre–post psychological factors (H1)

For each psychological factor, we compared the composite score from the baseline to the final and from the baseline to the follow-up. We ran a GLM in R [78], with factors time (baseline, final and follow-up) × condition (experimental and control).

#### 4.1.2. Pre–post behaviour (H2)

##### 4.1.2.1. H2a:

For each behaviour type (individual versus collective) and each behaviour category (civic participation, advocacy, education, activism, transportation, food, energy and finance), we compared the participants’ action scores tracked via EMA during the baseline, final and follow-up tracking period, between the experimental and control groups. To consider the breadth of the multi-day data (14 action scores per time period), we tested a linear mixed effect model via GLMER in R [78], with negative binomial or Poisson family distribution, based on the smallest Akaike information criteria, preserving the 14 repeated measures. This model included as predictors time (baseline–final and baseline–follow-up), condition (experimental and control), the time × condition interaction and participant as a random factor. To test the significance of the time × condition interaction, we used sequential ANOVA decomposition of fixed effects, comparing the main model to a model including all the same predictors except for the time × condition interaction.

##### 4.1.2.2. H2b:

We repeated the analyses from H2a to predict the plan score (i.e. the number of planned activities recorded for each day of the behavioural tracking) and the alignment score (i.e. the difference between actions performed and actions planned), from baseline to final and from baseline to follow-up. This was to assess whether the intervention improved the participants’ plans to take action and their ability to adhere to their plans.

##### 4.1.2.3. H2c:

For behavioural recall, entailing only one composite score per time point (baseline and follow-up), we ran a simple analysis of variance (ANOVA), with factors time (baseline and follow-up) × condition (experimental and control).

#### 4.1.3. Behaviour change as a result of psychological change (H3)

##### 4.1.3.1. H3a:

For each behaviour type and category that significantly changed in H2, we tested whether the change in the psychological factors predicted the mean final action score. We tested multiple linear regression models on the mean final action score, with the predictors: the mean baseline action score and the change (from baseline to final) in each psychological factor. We aggregated the daily action scores into a mean because H3 did not aim to examine day-to-day fluctuations in behaviour, but rather whether pre–post changes in psychological factors predicted overall changes in behavioural engagement from before to after the intervention. Note that only the change in the factors with a significant time × condition interaction from the H1 analysis was included. Because this hypothesis is not strictly related to the effectiveness of our intervention (as for H1 and H2), but rather to the general relationship between the psychological factors and behaviour, we did not include condition as a predictor. We ran the same model to predict the mean follow-up action score, for each behaviour type.

##### 4.1.3.2. H3b:

We repeated the same linear regression model to predict all behaviour types and categories for which the plan and alignment scores increased significantly in H2, with the predictors: the mean baseline plan score/alignment score and the change (from baseline to final) in each psychological factor.

### 4.2. Outliers

Outliers were excluded only when using parametric tests (e.g. for H1 ANOVA) and were identified using the box plot procedure [79] with the identify_outlier function in R. When outliers were found, we applied a 5% robust trimming method by removing the top and bottom 5% of the data from the respective analyses [80].

## 5. Results

### 5.1. Main quantitative results

#### 5.1.1. H1: psychological factors

McDonald's omega was > 0.7 for all psychological factors, confirming the questionnaires had at least moderate reliability (table 3). For the means and standard deviations of each factor at each time period, see the supplementary material, table S2. Four of the 12 factors had a significant time × condition interaction from baseline to final, where the factor score increased for the experimental group but not for control. This was the case for emotional engagement (*F*_1,74_ = 7.22, *p* = 0.009, *η*^2^ = 0.077)*,* collective efficacy (*F*_1,74_ = 5.33, *p* = 0.023, *η*^2^ = 0.060), bottom-up theory of change (*F*_1,80_ = 7.04*, p* = 0.009*, η*^2^ = 0.070) and appraisal (*F*_1,80_ = 5.96, *p* = 0.017, *η*^2^ = 0.061) (figure 2; table 3). The remaining factors did not show a significant time × condition interaction. The post hoc *t*-tests for each psychological factor are reported in table 3.

**Table 3.** Time × condition interactions of the 12 psychological factors. (Note: outliers were rejected using robust trimming. *p* < 0.05 is shown in bold.)

		baseline–final				baseline–follow-up			
		interaction		post hoc		interaction		post hoc	
**factor**	McDonald's *ω*	*p* (*F*)	*η*^2^	experimental *p* (*t*)	control *p* (*t*)	*p* (*F*)	*η*^2^	experimental *p* (*t*)	control *p* (*t*)
emotional engagement	0.89	**0.009** (7.22)	0.077	**<0.001** (5.32)	0.873 (–0.16)	**0.032** (4.89)	0.096	0.184 (1.39)	0.277 (–1.17)
self-efficacy	0.93	0.364 (0.83)	0.010			0.140 (2.27)	0.054		
collective efficacy	0.90	**0.023** (5.33)	0.057	**<0.001** (4.96)	0.382 (0.89)	**0.033** (4.80)	0.088	**0.008** (2.98)	0.331 (–1.03)
theory of change	0.84	**0.009** (7.04)	0.070	**0.001** (4.19)	0.948 (–0.06)	**0.020** (5.86)	0.128	**0.006** (3.29)	0.388 (–0.92)
cognitive alternatives	0.93	0.754 (0.10)	0.001			0.749 (0.10)	0.002		
controllability	0.77	0.599 (0.28)	0.003			0.704 (0.15)	0.003		
self-identity	0.88	0.706 (0.14)	0.002			0.951 (0.00)	0.000		
collective identity	0.90	0.836 (0.04)	0.000			0.233 (1.46)	0.034		
appraisal	0.90	**0.017** (5.96)	0.061	**0.001** (3.69)	0.735 (–0.34)	0.063 (3.62)	0.068		
trust in institutional politics	0.91	0.228 (1.47)	0.016			0.598 (0.28)	0.007		
implementation intentions	0.92	0.270 (1.23)	0.013			0.430 (0.64)	0.014		
social norm	0.70	0.321 (0.99)	0.011			0.208 (1.63)	0.034

**Figure 2.**
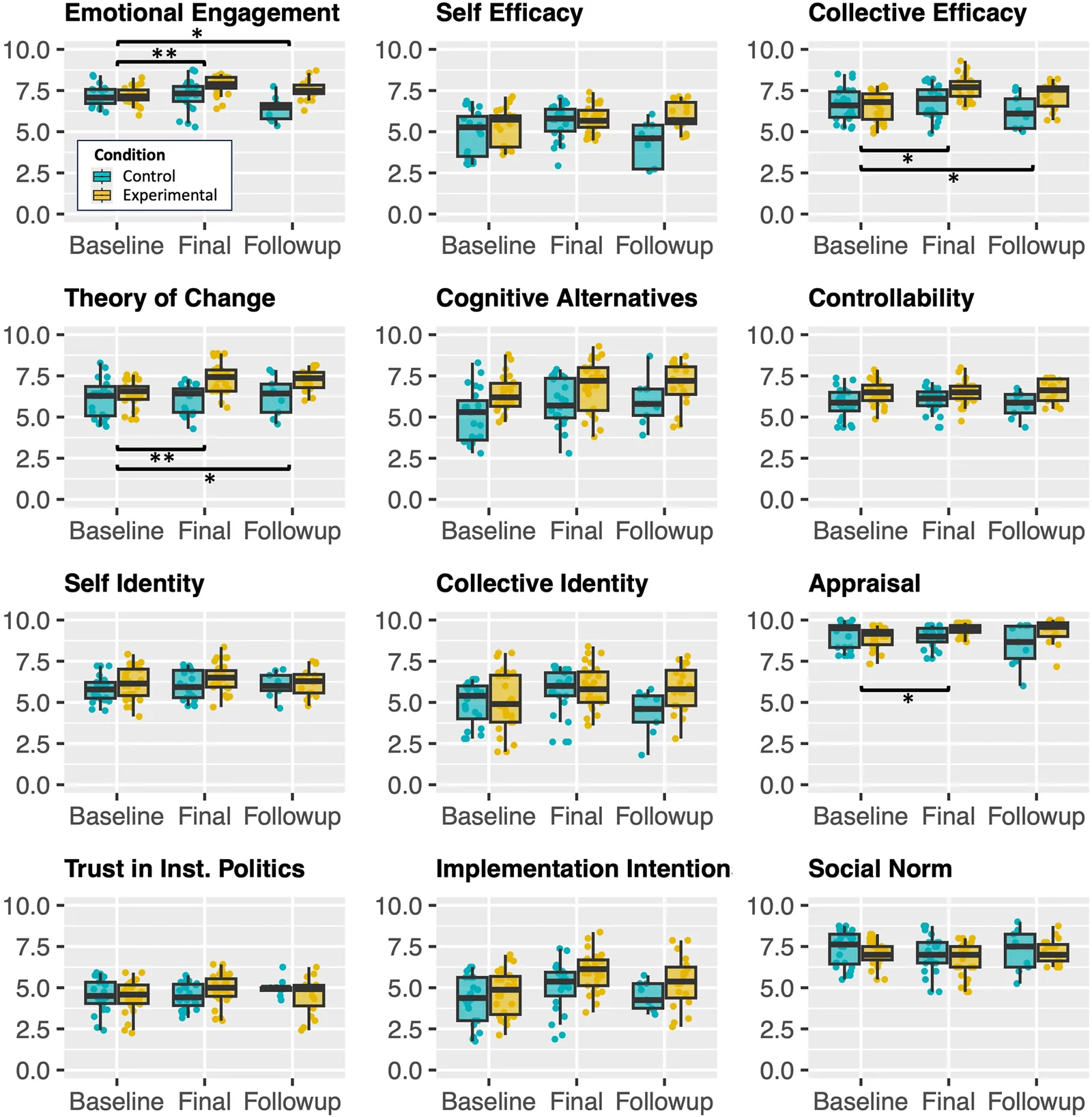
The 12 psychological factors by condition and time. Note*:* significant interactions are marked with horizontal brackets. Significant contrasts: **p < 0.01 and *p < 0.05.

For three of the factors, there was a significant time × condition interaction from baseline to follow-up. These were emotional engagement (*F*_1,42_ = 4.89, *p* = 0.032, *η*^2^ = 0.096), collective efficacy (*F*_1,50_ = 4.80, *p* = 0.033, *η*^2^ = 0.088) and bottom-up theory of change (*F*_1,40_ = 5.86, *p* = 0.020, *η*^2^ = 0.128) (figure 2; table 3). The post hoc *t*-tests showed that each of these factors increased for the experimental group and did not change significantly for the control group.

#### 5.1.2. H2: behavioural outcomes

For the baseline–final comparisons, we retained 30 experimental and 21 control participants who filled out at least five surveys at both time points (see §2.3. Exclusion criteria), for a total of *n* = 51. For the baseline–follow-up comparisons, we retained 23 experimental and 12 control participants, for a total of *n* = 35. The EMA group means and standard deviations of each behaviour score (action, planning and alignment) at each time point (baseline, final and follow-up) can be found in the supplementary material, table S3. Descriptive information about EMA submissions can be found in the supplementary material, table S4.

##### 5.1.2.1. Action score (H2a): collective:

For the aggregate collective action score, there was no significant time × condition interaction from baseline to final (figure 3A; table 4).

**Figure 3.**
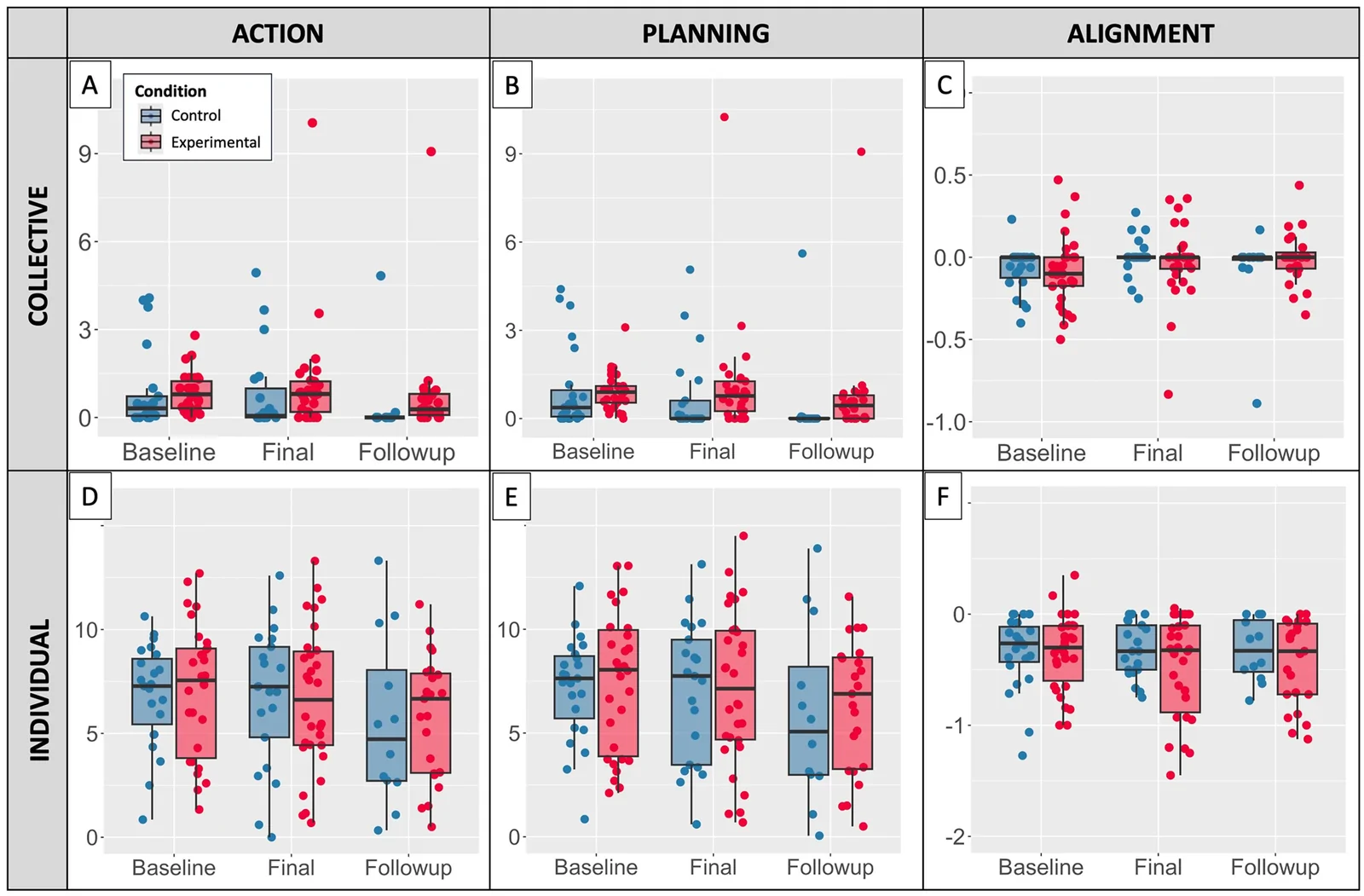
Change over time of the aggregate action (A,D), planning (B,E) and alignment (C,F) scores. Note*:* no significant time × condition interactions are present. For action and planning, the *y*-axis reports the mean number of daily actions per period (baseline, final and follow-up). For alignment, the *y*-axis reports the mean of the daily behaviour-plan score per period (see §2. Methods).

**Table 4.** GLMER model statistics of the time × condition interactions and post hoc tests of the aggregate EMA behavioural scores, that is action, planning and alignment.

	baseline–final			baseline–follow-up		
	interaction			interaction		
behaviour	estimate (CI)	s.e.	*p* (*z*)	estimate	s.e.	*p* (*z*)
collective ACTION	0.20 (–0.13 0.49)	0.15	0.171 (1.37)	0.26 (–0.37 0.75)	0.25	0.281 (1.08)
collective PLANNING	0.18 (–0.05 0.69)	0.15	0.237 (1.19)	0.29 (–0.18 0.94)	0.25	0.262 (1.12)
collective ALIGNMENT	0.01 (–0.09 0.12)	0.05	0.869 (0.164)	0.08 (–0.04 0.19)	0.06	0.217 (0.35)
individual ACTION	–0.09 (–0.24 0.02)	0.07	0.177 (–1.35)	–0.02 (–0.23 0.17)	0.08	0.775 (–0.29)
individual PLANNING	–0.06 (–0.20 0.08)	0.07	0.402 (–0.84)	0.03 (–0.12 0.25)	0.08	0.664 (0.43)
individual ALIGNMENT	–0.15 (–0.31 0.02)	0.08	0.074 (–1.76)	–0.11 (–0.32 0.10)	0.11	0.288 (–1.06)

However, when looking at the specific collective behavioural categories and considering the comparison between baseline and final, we found significant interactions for three behaviours (figure 4A; table 5): *civic participation* (*z* = 2.11, *p =* 0.034), which increased for experimental (*z =* 3.50, *p* < 0.001) and did not change for control (*z* = –0.14, *p* = 0.886); *advocacy* (*z* = 2.59, *p =* 0.009), which increased for experimental (*z =* 4.08, *p* < 0.001) and remained steady for control (*z* = 0.13, *p* = 0.899); and *activism* (*z* = 2.26, *p* = 0.020), which increased for experimental (*z* = 3.73, *p* < 0.001) and did not change for control (*z* = 0.14, *p* = 0.888).

**Figure 4.**
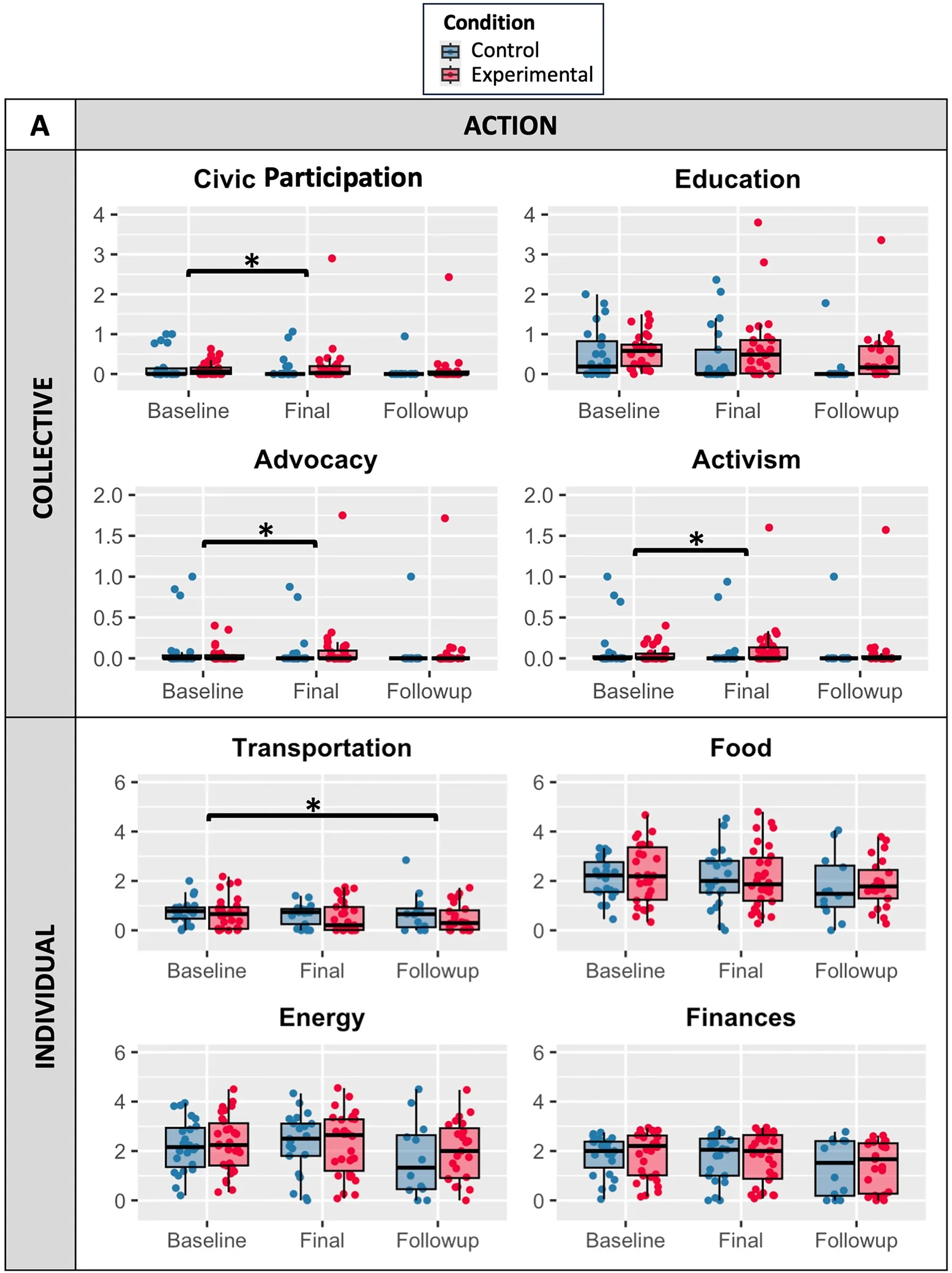

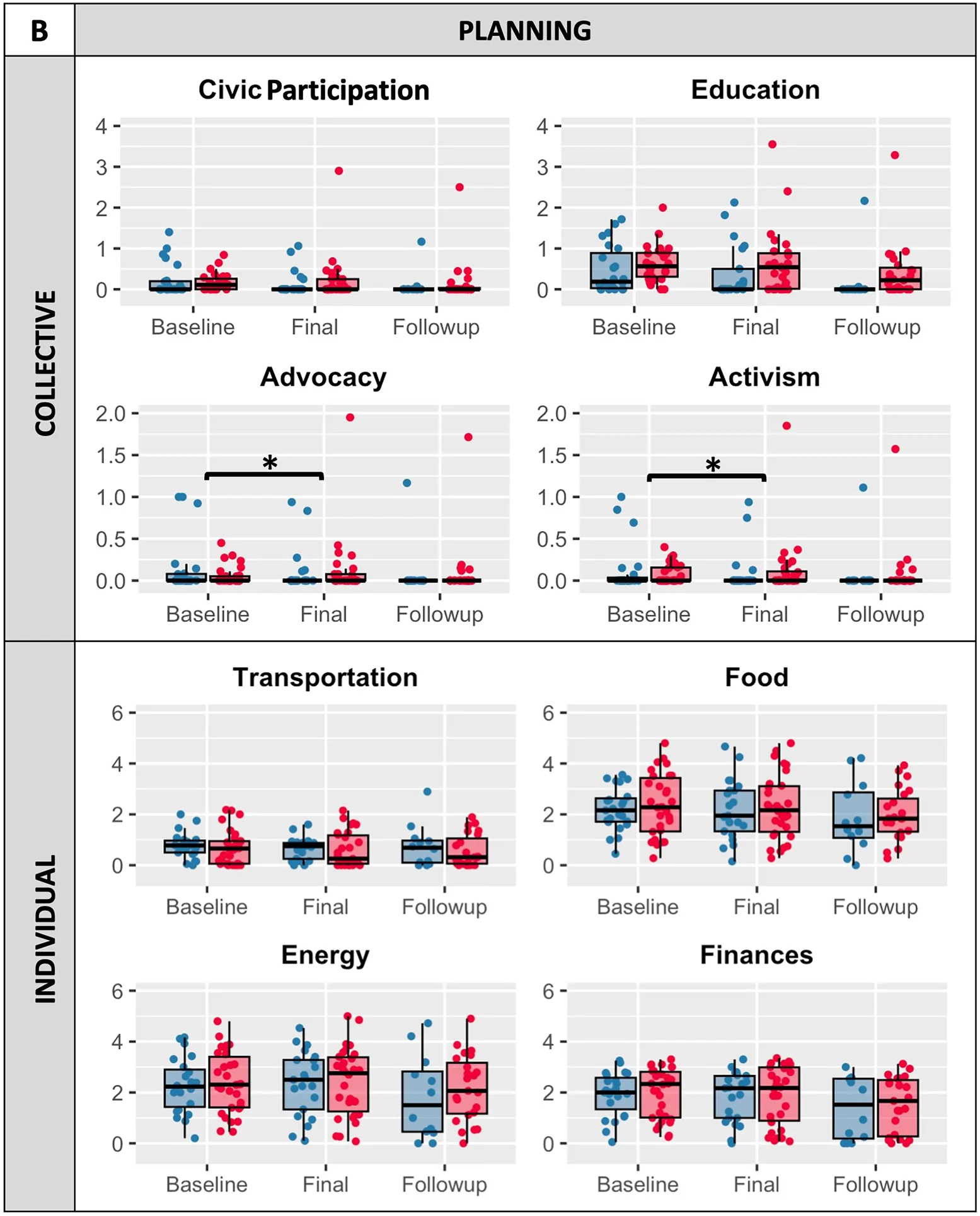

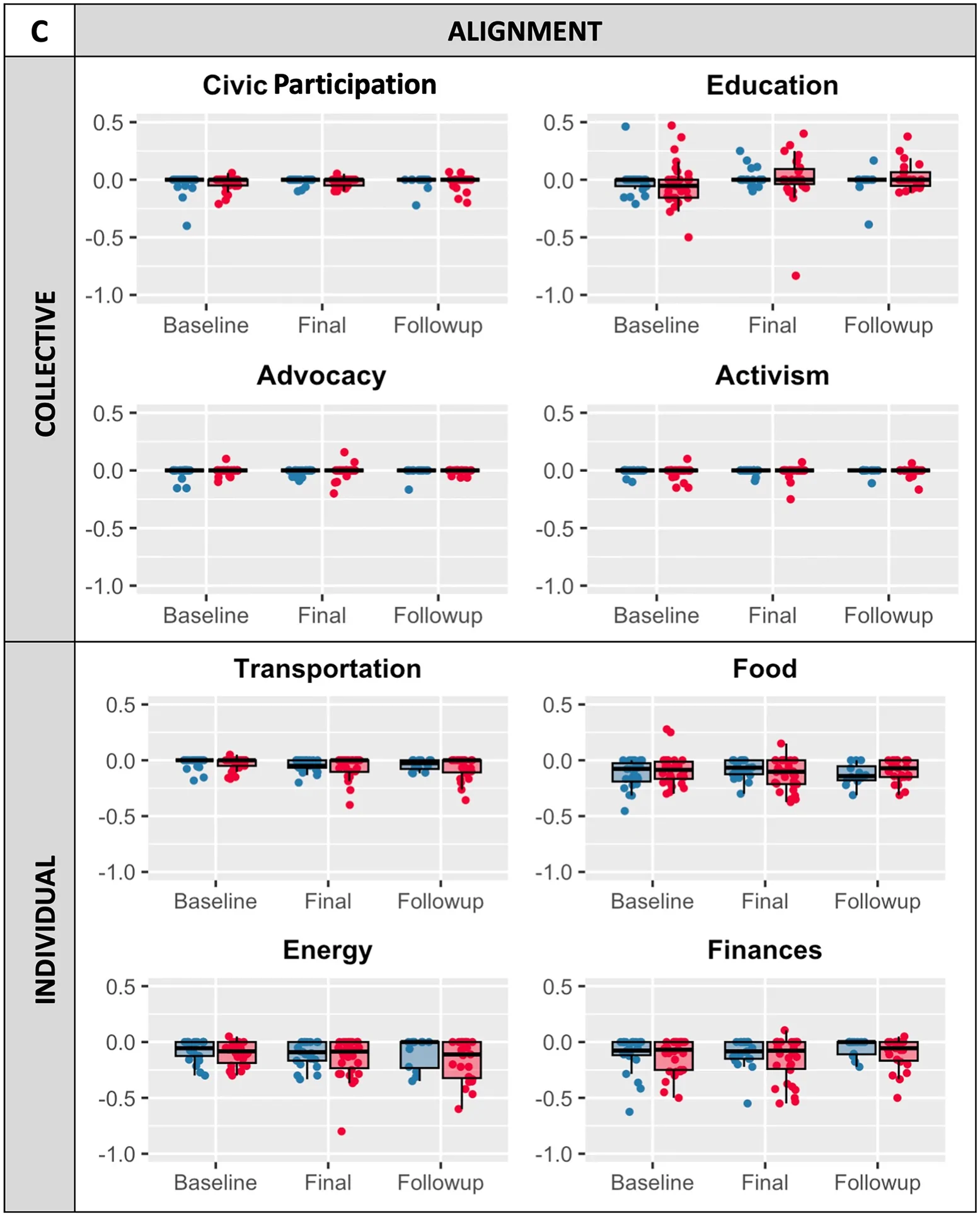
Change over time (baseline, final and follow-up) of the specific behavioural categories for the three behavioural scores, that is (A) action, (B) planning and (C) alignment. Note*:* bars indicate significant time × condition interactions, *p < 0.05.

**Table 5.** Significant time × condition interactions and post hoc tests by behavioural category. (Note: *p* < 0.05 shown in bold. For the complete set of statistics including non-significant interactions, see the supplementary material, table S5.)

	baseline–final				baseline–follow-up		
	interaction		post hoc		interaction	post hoc	
behaviour		*p* (*z*)	exp *p* (*z*)	cont *p* (*z*)	*p* (*z*)	exp *p* (*z*)	cont *p* (*z*)
collective action	civic participation	**0.034** (2.11)	**<0.001** (3.50)	0.886 (–0.14)	0.419 (0.81)		
	advocacy	**0.009** (2.59)	**<0.001** (4.08)	0.899 (0.13)	0.114 (1.58)		
	activism	**0.020** (2.33)	**<0.001** (3.79)	0.888 (0.14)	0.214 (1.24)		
collective planning	advocacy	**0.007** (2.70)	**<0.001** (4.21)	0.926 (0.09)	0.077 (1.77)		
	activism	**0.047** (1.99)	**<0.001** (3.39)	0.938 (0.08)	0.431 (0.79)		
individual action	transportation	0.308 (–1.02)			**0.021** (–2.31)	**0.006** (–2.73)	0.406 (0.83)

##### 5.1.2.2. Action score (H2a): individual:

The aggregate individual action score did not show any significant interaction for any of the time comparisons. However, the *transportation* category alone showed a significant interaction when comparing baseline and follow-up (*z* = –2.31, *p* = 0.021). This was because experimental participants decreased their use of sustainable transportation (*z* = –2.73, *p* = 0.006), and control did not change (*z* = 0.83, *p* = 0.406; see figure 4A; table 5).

##### 5.1.2.3. Planning score (H2b): collective:

For the aggregate collective planning score, there was no significant time × condition interaction from baseline to final (figure 3B; table 4), similarly to what we observed for the action score. However, when looking at the specific collective behavioural categories and the comparison between baseline and final, we found a significant interaction for *advocacy* planning (*z* = 2.70, *p* = 0.007), which increased significantly for experimental participants (*z* = 4.21, *p* < 0.001) and did not change for control (*z* = 0.09, *p* = 0.926); and for *activism* planning (*z* = 1.99, *p* = 0.047), which increased for experimental (*z* = 3.39, *p* < 0.001) and decreased for control (*z* = 0.08, *p* = 0.938) (figure 4B; table 5). There was no significant interaction when comparing baseline and follow-up.

##### 5.1.2.4. Planning score (H2b): individual:

The aggregate individual planning score did not show any significant interaction for any of the time comparisons (figure 4B; table 5).

##### 5.1.2.5. Alignment score (H2b):

For the collective and individual aggregate alignment scores, there was no time × condition interaction at any of the time points, nor there were significant interactions for the alignment scores of the specific collective and individual behavioural categories (figures 3C and 4C; tables 4 and 5).

##### 5.1.2.6. Behavioural recall (H2c):

There was no significant time × condition interaction for recall from baseline to follow-up, for either collective behaviour (*F*_1,98_ = 2.02, *p* = 0.157, *η*^2^ = 0.02) or individual behaviour (*F*_1,98_ = 1.48, *p* = 0.226, *η*^2^ = 0.01) (supplementary material, figure S2).

#### 5.1.3. H3: psychological factors predicting behaviour

Because the aggregate action, planning and alignment scores and 8 of the 12 factors targeted did not increase after the intervention, we only ran prediction models with the action, planning and alignment categories, as well as the psychological factors that did increase from baseline to final. We only considered the specific behavioural categories for which a significant time × condition interaction was found in the H2 analyses; namely *civic participation* (baseline versus final), *advocacy* (baseline versus final) and *activism* (baseline versus final). A summary of the results on all behavioural categories is reported in table 6. The only significant effect was for the *activism* action scores, where the increase in activism from baseline to final was significantly predicted by the increase in bottom-up theory of change (*β* = 0.06, s.e. = 0.03, *t*_47_ = 2.4, *p* = 0.019, *r* = 0.33).

### 5.2. Additional qualitative results

#### 5.2.1. Module diaries

Participants’ feedback on the six intervention modules revealed consistent themes. The most motivating and engagement-promoting elements were concrete examples of successful collective climate action, practical opportunities to plan and implement campaigns, and interactions with peers and climate activists. Less engaging elements primarily involved theoretical content, familiar information or activities perceived as difficult to translate into real-world action ( table 7).

#### 5.2.2. Focus group

In the focus group, participants shared their experiences in the intervention, mentioning what they found more and less inspiring, their experience with action engagement and their suggestions for optimizing the intervention. The results corroborated the quantitative findings and module feedback. Participants emphasized the motivation generated by the elements (materials and activities) of the intervention focusing on the impact of collective action, the opportunity to engage in practical actions in their daily life and the value of peer support in action engagement, while also identifying barriers to participation such as time constraints, logistical challenges and the perceived complexity of advocacy activities (table 8).

The results of the remaining additional analyses, that is climate anxiety and the change of the psychological factors across the intervention modules, can be found in the supplementary material.

## 6. Discussion

In this study, we replicated a six-week in-person educational intervention [25] designed to strengthen 12 psychological correlates of pro-climate action, with the goal of increasing both collective and individual climate action among university students in The Netherlands.

From before to after, we observed significant increases in four key psychological correlates of pro-climate behaviour, namely emotional engagement, collective efficacy, bottom-up theory of change and appraisal, compared to a control group. Three of these (all except appraisal) remained significantly higher at a three-month follow-up. Three collective behaviours increased significantly among intervention participants immediately after the programme, relative to control participants: civic participation (signing petitions, donating to climate campaigns or sporadically attending marches), advocacy (negotiating formally with politicians to introduce and apply climate mitigation policy) and activism (organizing grassroots initiatives to put pressure on politicians to enact climate mitigation policy). The latter two include considerable costs in terms of time, resources and exposure, and the fact that all three behaviours increased, even temporarily, is an encouraging result, given that such outcomes are rarely observed in similar interventions [13,25]. In their final interviews, participants reported that meeting real climate organizers during the intervention and joining a social media channel advertising activist events especially facilitated their engagement. Nevertheless, these behavioural increases faded out by follow-up. Several factors may explain this fade-out. First, the follow-up surveys were administered during the summer holidays, when students go on holiday. Second, international students expressed concerns about the risks of collective action in a foreign country, particularly in a context where climate activism is increasingly criminalized [81,82] and where political debates threaten international students’ access to higher education in The Netherlands [83,84]. Third, participants reported losing momentum once the intervention finished and the group separated. Future interventions might sustain engagement by providing participants with networking platforms.

**Table 6.** GLM models predicting the Action and Planning Scores of the specific categories. *Note. p* < 0.05 shown in bold. The predictors are the delta scores of the psychological factors significantly boosted by our intervention. We controlled by the baseline score of behaviour and by the baseline score of each psychological factor.

	civic engagement (Action) baseline–final						advocacy (Action) baseline–final					
	estimate	std. error	*t*	*p*	*df*	*r*	estimate	std. error	*t*	*p*	*df*	*r*
**emotional engagement**	0.05	0.06	0.72	0.476	47	0.10	0.02	0.04	0.60	0.553	47	0.09
**collective efficacy**	0.07	0.04	1.48	0.144	47	0.21	0.01	0.03	0.28	0.783	47	0.04
**theory of change**	0.05	0.05	1.05	0.299	47	0.15	0.02	0.03	0.84	0.404	47	0.12
**appraisal**	0.04	0.09	0.42	0.676	47	0.06	0.04	0.05	0.69	0.494	47	0.10
	**activism (Action) baseline–final**						**transportation (Action) baseline–final**					
	**estimate**	**std. error**	***t***	***p***	***df***	***r***	**estimate**	**std. error**	***t***	***p***	***df***	***r***
**emotional engagement**	0.05	0.04	1.33	0.191	47	0.19	–0.04	0.05	–0.72	0.478	47	0.10
**collective efficacy**	0.05	0.03	1.99	0.053	47	0.28	–0.06	0.04	–1.55	0.128	47	0.22
**theory of change**	0.06	0.03	2.42	**0.019**	47	0.33	–0.02	0.04	–0.44	0.665	47	0.06
**appraisal**	0.08	0.05	1.60	0.117	47	0.23	–0.04	0.07	–0.53	0.596	47	0.08
	**advocacy (Planning) baseline–final**						**activism (Planning) baseline–final**					
	**estimate**	**std. error**	***t***	***p***	***df***	***r***	**estimate**	**std. error**	***t***	***p***	***df***	***r***
**emotional engagement**	0.03	0.05	0.60	0.553	47	0.09	0.05	0.04	1.31	0.197	47	0.19
**collective efficacy**	0.02	0.03	0.58	0.566	47	0.08	0.04	0.03	1.46	0.151	47	0.21
**theory of change**	0.03	0.03	1.00	0.321	47	0.14	0.04	0.03	1.44	0.156	47	0.21
**appraisal**	0.03	0.06	0.49	0.625	47	0.07	0.06	0.06	1.09	0.280	47	0.16

**Table 7.** Summary of the participants’ feedback on the six intervention modules. (Note: a more comprehensive version of this table is provided in the supplementary material, table S7.)

topic	most frequently mentioned themes
motivating/interesting elements	examples of successful climate action campaigns and social movements, practical campaign planning, interactions with peers and climate activists and understanding how societal change can occur starting from people
demotivating/boring elements	familiar climate change information, highly theoretical content and the lack of time to implement the planned campaigns
helpful for engagement	learning that collective action can drive meaningful change, concrete advocacy and organizing strategies, information about climate organizations and opportunities for participation

**Table 8.** Summary of the focus group results. (Note: a more comprehensive version of this table is provided in the supplementary material, table S8.)

topic	main themes
most inspiring elements	learning about the potential impact of collective action and successful campaigns, practical action planning tools and learning/action support from other intervention participants
least inspiring elements	activities perceived as overly theoretical and campaign-building tasks that felt complex or difficult to implement
behavioural changes	increased engagement in collective action, climate-related learning, peer education and some individual lifestyle changes
main barriers to action	time constraints, costs, logistical barriers and the effort required for advocacy and activism
main facilitators of action	social support from peers, learning about local opportunities for collective participation and actions that fit existing habits and routines
suggestions for improvement	less theory, more practical implementation of campaigns, more opportunities for real-world action and continued engagement after the intervention

The increase in activism was predicted by increases in the bottom-up theory of change, which reflects the belief that bottom-up initiatives can scale up to produce systemic change [51,52]. A bottom-up theory of change may be particularly relevant for activism, where top-down institutional solutions are often questioned and greater emphasis is placed on the capacity of grassroots organizing to drive change from the bottom. While this construct may also underpin other forms of collective action, which we may have lacked sufficient power to detect, civic participation, as operationalized in this study, involves less strategic planning for change (e.g. individuals simply attend events without engaging in their organization or strategizing their impact). Advocacy, in turn, generally relies more on trust in formal, top-down policy-making processes. Therefore, it is not surprising that, even if the latter two forms of engagement increased through the intervention, they were not directly associated with increases in the bottom-up theory of change.

The significant effects observed were generally consistent with the effect sizes anticipated during study planning. H1 yielded small-to-moderate effects that closely matched the pre-registered smallest effect size of interest (*f²* = 0.09), while the significant H2 effects derived from the interaction *z*-statistics ranged from small to moderate and included effects near the pre-registered expectation (*d* = 0.25). For H3, associations ranged from negligible to moderate, with the strongest effects approaching the expected magnitude (e.g. theory of change predicting activism, *f²* = 0.12 versus the pre-registered *f²* = 0.15).

Unexpectedly, sustainable transportation decreased by follow-up among intervention participants compared to controls. This may be explained by the timing of the follow-up surveys: during holidays, students may have travelled to cities less suited for biking than Amsterdam and possibly travelled by car or plane. In such contexts, the participants who attended the intervention may have put less effort into finding ways to travel sustainably, having shifted their focus from individual to collective action. In fact, in the focus group and module diaries, experimental participants mentioned being especially galvanized by collective actions after realizing their greater impact compared to individual engagement alone.

The qualitative feedback showed that participants were particularly motivated by the power of success stories of past collective movements (such as the civil rights movement or the movement against ozone depletion). They valued learning how small campaigns can grow into large-scale movements and appreciated the practical guidance on campaign building. However, they were disappointed not to implement their planned campaigns. Future studies should test the psychological and behavioural impact of making participants enact real-world initiatives. Participants also expressed interest in learning more about personal benefits of climate action (e.g. mental health, social networking and skill development), which they found particularly motivating, and preferred activities with tangible impact over purely psychological exercises. For instance, campaign building (aimed at boosting collective efficacy) was motivating because of its applied relevance, while activities, such as sharing emotions or values (aimed at leveraging emotional engagement and self-identity), were perceived as less impactful. This suggests that interventions aiming to leverage psychological factors should balance psychological stimulation and opportunities for visible, real-world impact.

The study required a substantial time commitment from participants, including six in-person 3-hour sessions with additional homework, six weeks of daily behavioural tracking and completion of three psychological questionnaires. Given the modest incentives offered (a certificate and small monetary rewards), participation was expected to attract primarily highly motivated individuals already inclined towards climate action (though not yet active, as ensured by the screening criteria). Although this self-selection limits the generalizability of the findings, it enabled the study to focus on individuals who were concerned about climate change but uncertain about how to translate that concern into concrete action. Large-scale surveys (e.g. [85,86]) indicate that the majority of people worldwide are concerned about climate change and motivated to act, yet remain behaviourally inactive. We see it as a priority to mobilize this alarmed but inactive majority before focusing on those who are unaware of, or deny, the climate crisis.

Although this intervention involved a smaller-than-expected number of participants, it drove relevant effects on several psychological and behavioural outcomes, raising the question of how similar interventions could be scaled to larger audiences. Monetary rewards may not always be feasible at larger scales, and the substantial time commitment required by the present intervention may limit participation if implemented exclusively as a voluntary activity. However, similar programmes may be integrated into existing for-credit educational or professional training structures. Future research should examine the feasibility and effectiveness of such implementations.

Our study had the following main limitations. First, the smaller-than-planned sample size substantially reduced our power to detect potential effects. Consequently, the absence of statistically significant effects may reflect limited statistical sensitivity (as suggested by supplementary material, table S6) rather than the absence of meaningful effects. Indeed, several observed effects were small to moderate in magnitude despite failing to reach conventional significance thresholds. Additionally, the low power may have limited the precision of effect estimates. The smaller-than-planned sample size was owing to a combination of recruitment challenges and higher-than-expected attrition throughout the study, including during both the intervention and follow-up phases. These challenges probably stemmed from the intensive nature of the intervention, which combined in-person sessions with repeated questionnaires and high frequency EMA participation over multiple weeks. Compared to many intervention studies on climate engagement, which often rely on shorter interventions, single-session manipulations or cross-sectional assessments, the present study required substantially greater sustained participant commitment over time. Additionally, the study offered relatively modest compensation given the time and effort required. We suggest that future studies provide stronger incentives, ideally integrating interventions into school, university or workplace credit systems. Second, we employed a mixed active–passive control group rather than a fully active one, given the difficulty of recruiting students already enrolled in environment-related courses. These students may have considered attending an additional climate programme excessive, on top of their already environment-related coursework. While this may be resource-intensive, future research should aim to design an alternative intervention for a fully active control group. Third, the daily EMA format may have been less sensitive to capturing lower-frequency collective behaviours compared to more routine individual actions. In particular, the behavioural categories involving structurally demanding actions that may not occur on a daily basis, such as civic engagement, advocacy and activism, showed substantial floor effects, possibly reflecting a mismatch between the temporal scale of these behaviours and the daily measurement format. However, these behaviours were included because even infrequent actions may represent meaningful climate engagement, and daily assessments allowed us to capture increases in their occurrence and planning over the EMA periods. Nevertheless, future studies may consider integrating longer-term assessments for capturing less frequent forms of climate action. Finally, our behavioural tracking (EMA) relied on self-reports rather than objective indicators. While EMA has notable strengths compared to intentions and recall [22,87] and has been used effectively in prior research (e.g. [25,88–90]), self-reports remain vulnerable to social desirability bias [91]. Although comparisons with the control group should mitigate this issue, future studies should incorporate more objective measures to strengthen causal claims about real-world behaviour.

## 7. Conclusion

An educational intervention strengthened key psychological factors related to climate engagement, namely bottom-up theory of change, collective efficacy, emotional engagement and appraisal, and increased engagement in collective climate action. These findings underscore the potential of longitudinal and manipulation-based studies to gain insights into the psychological motivators of collective climate action. In particular, the link between the increase in bottom-up theory of change and activism points to the importance of people's motivation to act from the bottom to reach societal transformations. The fade-out of collective actions by three months highlights that long-term engagement needs structural support that goes beyond educational materials, such as networking platforms, collaborations with real-world climate organizations and practical information about local collective action initiatives to help maintain momentum. Optimizing this intervention could ultimately lead to a comprehensive set of didactic materials for climate action education programmes targeting young adults. In summary, despite the tendency of many climate change education programmes to elicit anxiety, disempowerment [92] or apathy rather than action [93], this intervention shows how environmental psychology can inform interactive educational designs that empower participants and promote collective action.

## Disclaimer

This Registered Report was submitted to *Royal Society Open Science* following peer review and recommendation for stage 2 acceptance at the Peer Community In (PCI) Registered Reports’ platform. Full details of the peer review and recommendation of the paper at PCI Registered Reports may be found at the link below.

After submission to the journal, the paper received no additional external peer review but was accepted on the basis of the Editor's recommendation according to our PCI Registered Reports’ policy.

Stage 1 and stage 2 recommendation and review history are available at https://rr.peercommunityin.org/articles/rec?id=1560.

## Supplementary Material



## Data Availability

All anonymized data and codes are available at the Open Science Framework: https://osf.io/9kve7/files/osfstorage?view_only=02aafa977bd64a2aac6158d33b4e87f3. Supplementary material is available online [94].
