# Supplementary material for: A climate action intervention increases four psychological motivators, as well as climate advocacy, activism and civic participation
Source: R Soc Open Sci. 2026 Oct 21;13(10):261346. doi: 10.1098/rsos.261346 (PMC13682933; doi:10.1098/rsos.261346)
Supplement: Supplementary file 1 [file rsos.261346.zip › rsos.261346/rsos261346_si_002.docx]

**Supplementary Materials**

**Table of contents:**

- Screening survey
- Baseline survey
  - Psychological questionnaires: 12 factors (baseline, final, follow-up)
  - Anxiety (baseline, final, follow-up)
  - Behavioral Recall
  - Demographics
- Ecological Momentary Assessment and Coding
- Final survey
  - Psychological questionnaires: 12 factors (baseline, final, follow-up)
  - Anxiety (baseline, final, follow-up)
- Follow-up survey
  - Psychological questionnaires: 12 factors (baseline, final, follow-up)
  - Anxiety (baseline, final, follow-up)
  - Behavioral Recall
- Focus group
- Intervention content table: modules title, factors targeted, and activity description
- Intervention measures:
- Comprehension questions
- Short psychological questionnaires
- Module diary
- Example module 1: factors + contents and activities
- Incentives system and exclusion
- Additional figures and tables
- Deviations from pre-registered study
- Sensitivity Analysis

—------------------------------------------------------------------------------------------------------------

**SCREENING SURVEY**

*Beliefs about Climate Change:*

1. Regardless of its cause, I am certain that climate change is actually occurring. Yes, No
2. Human activities are a significant cause of climate change. Yes, No

*Additional selection criteria:*

1. Are you between 18 and 15 years of age? Yes, No
2. Are you proficient in English? Yes, No
3. Have you participated in at least three climate-related civic initiatives in the past three months, for example, organized/attended climate-related political or educational campaigns, demonstrations, protests, petitions, civil disobedience actions, or others? Yes, No

**BASELINE SURVEY**

***12 psychological factors***

1. Emotional engagement (modified from Howe et al., 2015, Leiserowitz et al., 2006; Rees et al., 2015; Landmann & Naumann., 2024)
   1. How worried are you about greenhouse gas emissions in general? From 1(not at all) to 10 (very much)
   2. How much do you think greenhouse gas emissions will harm plants and animal species:
      1. Globally ? From 1 (not at all) to 10 (very much)
      2. In your region? From 1 (not at all) to 10 (very much)
   3. How much do you think greenhouse gas emissions will harm future generations:
      1. Globally ? From 1 (not at all) to 10 (very much)
      2. In your region? From 1 (not at all) to 10 (very much)
   4. How much do you think greenhouse gas emissions will harm:
      1. People globally?
      2. People in developing countries? From 1 (not at all) to 10 (very much)
      3. You? From 1 (not at all) to 10 (very much)
   5. When do you think greenhouse gas emissions will begin to harm:
      1. People around the world? [reversed] 1 (It is already happening), 2 (in the next 10 years), 3 (in the next 100 years), 4 (in the next 100 years), 5 (in more than 100 years)
      2. People in your region? [reversed] 1 (It is already happening), 2 (in the next 10 years), 3 (in the next 100 years), 4 (in the next 100 years), 5 (in more than 100 years)
      3. You personally? (It is already happening), 2 (in the next 5 years), 3 (in the next 10 years), 4 (in the next 20 years), 5 (in more than 20 years)
   6. Many people’s standard of living will decrease due to greenhouse gas emissions:
      1. Worldwide 1 (Not at all likely) to 10 (Highly likely)
      2. In your region 1 (Not at all likely) to 10 (Highly likely)
   7. There will be an increase in extreme weather events in the world due to greenhouse gas emissions. 1 (Not at all likely) to 10 (Highly likely)
   8. Rate how much risk there is that greenhouse gas emissions will reduce your personal well-being in the following ways, from 1 (No risk) to 10 (High risk):
      1. By increasing extreme weather events in your area
      2. By politically destabilizing your region
      3. Causing job loss in your region
      4. Forcing you to migrate
      5. Reducing the resources available in your region (such as water, food, services, etc.)
   9. When thinking about the climate crisis, I feel: From 1 (not at all) to 10 (very much):
      1. Guit
      2. Sadness
      3. Fear
      4. Shame
      5. Anger
      6. Emotional coldness
      7. Empathy for those affected by climate impacts
      8. Hope
      9. Connection with the people working on mitigation

2. Self-efficacy (modified from Hamann et al., 2020; Gulliver et al., 2022)
1. Changes in my daily routines (from diet, energy use, transports and finances) can contribute to mitigate climate change. From 1 (Completely Disagree) to 10 (Completely Agree)
2. I don’t think that I as an individual can encourage others to [reversed]: From 1 (Completely Disagree) to 10 (Completely Agree)
   1. Reduce their personal emissions (from diet, energy, transports and finances)
   2. Engage in collective pro-climate behaviors (such as civic participation, climate education, advocacy and activism)
3. My individual pro-climate actions will encourage others to do the same. From 1(not at all) to 10 (very much)
4. My collective pro-climate actions will encourage others to do the same. From 1(not at all) to 10 (very much)
5. I think that my skills and competences can help a climate group/organization be successful. From 1(not at all) to 10 (very much)
6. To which extent do you feel capable of: From 1(not at all) to 10 (very much)
   1. Distributing information about a climate group/organization’s cause
   2. Organizing a local event with a climate group/organization
   3. Doing volunteer tasks for a climate group/organization outside of meetings
   4. Volunteering in the role of a committee member, coordinator or similar for a climate group/organization
   5. Giving a presentation or talk on a climate issue
   6. Recruiting new volunteers for a climate group/organization
   7. Starting a new environmental project, campaign, program or group/organization
   8. Confronting public representatives about a climate issue
   9. Organizing a protest/demonstration with a climate group/organization

3. Collective efficacy (modified from Hamann et al., 2020; Gulliver et al., 2022)
1. I think that our joint actions as students can contribute to mitigating the climate crisis, by reducing greenhouse gas emissions. From 1 (not at all) to 10 (very much)
2. I don’t think that we as students can encourage others to promote emission-reduction behaviors. [reversed] From 1 (Completely Disagree) to 10 (Completely Agree)
3. Our pro-climate actions as students will encourage others to do the same. From 1 (not at all) to 10 (very much)
4. Together people who volunteer in a climate group/organization can achieve relevant emission reduction goals. From 1 (Completely Disagree) to 10 (Completely Agree)
5. Efforts to volunteer for the climate in a group/organization in my city are a waste of time. From 1 (Completely Disagree) to 10 (Completely Agree)
6. A group of climate advocates/activists can convince a local administration to enact climate policy. From 1 (not at all) to 10 (very much)
7. A climate group/organization can use effective strategies (identify clear goals and targets, use effective tactics, plan timelines) to achieve emission reduction goals. From 1 (not at all) to 10 (very much)
8. Regardless of the strategy they use, climate groups/organizations are unlikely to reach relevant mitigation goals. [reversed] From 1 (Completely Disagree) to 10 (Completely Agree)
9. A solid internal structure (distribution of roles, responsibilities and leadership) can make a climate group/organization particularly effective at achieving emission reduction goals. From 1 (not at all) to 10 (very much)
10. Regardless of their internal structure, climate groups/organizations are unlikely to reach relevant mitigation goals. [reversed] From 1 (Completely Disagree) to 10 (Completely Agree)

4. Bottom-Up Theory of change (modified from Castiglione et al., 2022)

       From 1 (Completely Disagree) to 10 (Completely Agree):

1. Climate mitigation can start locally (e.g. in my city, in my school, in my company) and spread globally.
2. Reducing emissions at one place (e.g. in my city, in my school, in my company) creates a precedent for similar places to do the same.
3. If a group of people puts pressure on a local administration to cut emissions, and another group does the same in another place, little by little we can create global change.
4. Reducing emissions in one place (e.g. in my city, in my school, in my company) is completely useless because big polluters somewhere else are countering this effort. [reversed]
5. Climate mitigation can start with common people asking/putting pressure on their local administrations to cut emissions.
6. Climate mitigation can only start with governments enacting emission reduction policies from the top. [reversed]
7. Voting is the only way that a person like me can have any say about climate policy. [reversed]

5. Cognitive alternatives (Wright et al., 2020)

From 1 (Completely Disagree) to 10 (Completely Agree):

1. I can easily imagine a world in which we supply all of our energy needs without harming the natural world.
2. It is easy to imagine a world where we no longer use fossil fuels.
3. I can clearly imagine a world where we exclusively use agricultural practices that protect the natural habitats of animals.
4. I can imagine a world where politicians care more about minimizing the population's negative impact on the environment than economic growth.
5. I can think of numerous methods of achieving a world where carbon emissions are reduced below current levels.
6. I can easily imagine a world where people see themselves as integrated with nature, rather than masters over the natural world.
7. When I imagine what an ecologically sustainable existence for humans would be like, I can picture it in detail.
8. A harmonious relationship between humans and the natural world is easy for me to imagine.
9. I can easily imagine a city/neighborhood/company that cut all financial ties with the fossil fuel industry.
10. I can easily imagine a fully decarbonized city/neighborhood/company.

6. Controllability (modified from Fielding et al., 2008)
1. How much control do you have over whether you:
   1. Reduce your personal emissions (from diet, transports, energy and finances)? From 1 (not at all) to 10 (very much)
   2. Engage in collective climate action (such as civic participation, education, advocacy, activism)? From 1 (not at all) to 10 (very much)"
2. How difficult would it be for you to:
   1. Reduce your personal emissions (from diet, transports, energy and finances)? [reversed] From 1 (not at all difficult) to 10 (very difficult)
   2. Engage in collective climate action (such as civic participation, education, advocacy, activism)? From 1 (not at all difficult) to 10 (very difficult)
3. It is mostly up to me whether I:
   1. Reduce my personal emissions (from diet, transports, energy and finances). From 1 (Completely Disagree) to 10 (Completely Agree)
   2. Engage in collective climate action (such as civic participation, education, advocacy, activism)? From 1 (Completely Disagree) to 10 (Completely Agree)

7. Implementation Intentions (modified from Gollwitzer & Sheeran, 2006)
1. I have plans for when, where and how I will: From 1 (I have no plan) to 10 (I have a detailed plan)
   1. reduce my energy carbon footprint
   2. reduce my transportation carbon footprint
   3. reduce my transportation carbon footprint
   4. reduce my finance carbon footprint
   5. engage civically to vote, sign petitions or support campaigns in favor of a climate issue
   6. educate others on pro-climate behaviors they can engage with
   7. engage in climate advocacy
   8. engage in climate activism

8. Social norm (modified from Fielding et al., 2008)

From 1 (Completely Disagree) to 10 (Completely Agree)

1. Young adults like me are already reducing their personal emissions (from diet, energy, transportation, and finances).
2. Young adults like me are already engaging collectively to mitigate climate change (engaging civically, educating others, doing advocacy or activism).

From 1 (Completely Disapprove) to 10 (Completely Approve)

1. Most people who are important to me (friends, family, peers) would approve/disapprove if I reduced my personal emissions (from diet, energy, transportation, shopping).
2. Most people who are important to me (friends, family, peers) would approve if I engaged collectively to mitigate climate change (engaging civically, educating others, doing advocacy or activism).

9. Self-Identity (modified from Mayer & Frantz, 2004; Plant & Devine, 1998)
1. I attempt to engage in collective pro-climate behaviors (such as civic participation, climate education, advocacy and activism) because it is personally important to me. From 1 (Completely Disagree) to 10 (Completely Agree)
2. I attempt to engage in individual pro-climate behaviors (reducing my emissions from diet, energy use, transportation and shopping) people because it is personally important to me. From 1 (Completely Disagree) to 10 (Completely Agree)
3. According to my personal values, living a high-carbon lifestyle is OK. [inverted] From 1 (Completely Disagree) to 10 (Completely Agree)
4. According to my personal values, NOT engaging with other citizens to mitigate the climate crisis is OK. [inverted] From 1 (Completely Disagree) to 10 (Completely Agree)
5. Living a low-carbon lifestyle is important to my self-concept. From 1 (not at all) to 10 (very much)
6. Engaging collectively with other citizens to mitigate the climate crisis is important to my self-concept. From 1 (not at all) to 10 (very much)
7. I think of the natural world as a community to which I belong. From 1 (not at all) to 10 (very much)
8. I often feel disconnected from nature. [reversed] From 1 (not at all) to 10 (very much)
9. When I think of my life, I imagine myself to be part of a larger cyclical process of living. From 1 (Completely Disagree) to 10 (Completely Agree)
10. I often feel a kinship with animals and plants. From 1 (not at all) to 10 (very much)
11. I have a deep understanding of how my actions affect the natural world. From 1 (Completely Disagree) to 10 (Completely Agree)
12. I feel that all inhabitants of Earth, human, and nonhuman, share a common ‘life force’. From 1 (not at all) to 10 (very much)
13. When I think of my place on Earth, I consider myself to be a top member of a hierarchy that exists in nature. [reversed] From 1 (Completely Disagree) to 10 (Completely Agree)
14. My personal welfare is independent of the welfare of the natural world. [reversed] From 1 (Completely Disagree) to 10 (Completely Agree)

10. Collective Identity (modified from Brick &Lai, 2018)
1. I see myself as an environmentalist. From 1 (Completely Disagree) to 10 (Completely Agree)
2. I feel strong ties with environmentalists. From 1 (Completely Disagree) to 10 (Completely Agree)
3. I identify with other environmentalists. From 1 (not at all) to 10 (very much)
4. I see myself as part of a community of people, reducing their personal greenhouse gas emissions to mitigate climate change. From 1 (not at all) to 10 (very much)
5. I see myself as part of a community of civically active people, who are participating in collective climate mitigation initiatives (such as climate advocacy, climate activism and climate education). From 1 (not at all) to 10 (very much)

11. Appraisal (modified from [Nordlund et al., 2003](https://www.sciencedirect.com/science/article/pii/S0272494403000379))
1. I am aware of the sources, impacts and solutions of climate change. From 1 (Completely Disagree) to 10 (Completely Agree)
2. A small increase in global temperatures leads to more catastrophic extreme weather events. From 1 (not at all) to 10 (very much)
3. It is a serious problem that increasing global temperatures are leading to more catastrophic extreme weather events. From 1 (Completely Disagree) to 10 (Completely Agree)
4. The increasing global temperature is threatening our way of living as we know it today. From 1 (not at all) to 10 (very much)
5. We need to urgently decarbonize our society, in order to mitigate climate change. From 1 (not at all) to 10 (very much)
6. Some core financial, economic, political, and social mechanisms in our society are preventing true emission reductions. From 1 (not at all) to 10 (very much)

12. Trust in institutional politics (modified from Miller & Miller, 1980; Saikkonen & Christensen,., 2023)
1. Elected representatives quickly forget the worries of ordinary citizens on climate change. [reversed] From 1 (Completely Disagree) to 10 (Completely Agree)
2. Citizens’ opinions on climate change are considered in political decision-making. From 1 (Completely Disagree) to 10 (Completely Agree)
3. Politicians do not care about the opinions of ordinary people regarding climate change. [reversed] From 1 (Completely Disagree) to 10 (Completely Agree)
4. I believe that some politicians want to enact true emission reduction policy. From 1(not at all) to 10 (very much)
5. Politicians don't care about climate change nor want to do anything to mitigate it. [reversed] From 1 (Completely Disagree) to 10 (Completely Agree)
6. I believe that politicians will enact climate mitigation policy, if asked to do so by the people. From 1 (Completely Disagree) to 10 (Completely Agree)
7. People like me don't have any say about what the government does on climate change. From 1 (Completely Disagree) to 10 (Completely Agree)
8. Sometimes politics and government seem so complicated that a person like me can't really do anything to influence climate policy. [reversed] From 1 (Completely Disagree) to 10 (Completely Agree)

***Climate Anxiety***

Modified from Climate anxiety scale (Clayton and Karazsia, 2020). All items: From 1 (Completely Disagree) to 10 (Completely Agree)

1. Thinking about climate change makes it difficult for me to concentrate.
2. Thinking about climate change makes it difficult for me to sleep.
3. I have nightmares about climate change
4. I find myself crying because of climate change
5. I think, “why can't I handle climate change better?”
6. I write down my thoughts about climate change and analyze them
7. I think, “why do I react to climate change this way?”
8. My concerns about climate change make it hard for me to have fun with my
   family or friends.
9. My concerns about climate change interfere with my ability to get work or
   school assignments done.
10. My concerns about climate change undermine my ability to work to my
    potential.
11. My friends say I think about climate change too much.

***Behavioral recall***

In the past 3 months, how often did you do the following?

Rate: 1) Never, 2) about once a month, 3) about once a week, 4) about twice a week, 5) every day:

1. Reduce your diet carbon footprint (e.g. by limiting meat or dairy consumption or only eating local products)?
2. Reduce your transportation carbon footprint (e.g. by limiting car use or plane travel and favoring biking, public transports or walking)?
3. Reduce your energy use carbon footprint (e.g. by unplugging appliances, switching lights off, limiting hot water use)?
4. Reduce your finances carbon footprint (reducing the amount of non-essential goods you buy, or avoiding investing your money in fossil-related products and services)?
5. Engage civically to vote, sign petitions, support campaigns in favor of a climate issue or join a demonstration?
6. Educate others on the causes and impact of the climate crisis, and pro-climate behaviors they can do to mitigate it?
7. Engage in climate advocacy by contacting and negotiating with local administrations/institutions to enact emission reduction policies?
8. Engage in climate activism by organizing grassroots events to put pressure on local administrations/institutions to enact emission reduction policies?

***Demographics***

1. How old are you?
2. Gender: Male, Female, Other (specify), Prefer not to say
3. What is your education level? Highschool diploma, Bachelor degree, Master’s degree doctorate degree
4. What is your income? Less than 10,000, 10,000-20,000, 20,000-30,000, 40,000-50,000, 50,000-60,00….140,000-150,000, 150,000 or more.
5. What is your political orientation? Right, Left, Center, Apolitical
6. In which country were you born?
7. In which country did you primarily grow up?

**ECOLOGICAL MOMENTARY ASSESSMENT AND CODING**

Every evening for two weeks, participants filled out a 10-minute survey, composed of two parts: 1) actions performed today and 2) action plan for tomorrow. Under individual and collective behaviors, they selected up to 5 behaviors, for each of 4 behavioral categories. The behavioral categories for individual behaviors were: 1. energy conservation, 2. food consumption, 3. transportation, 4. finances. The behavioral categories for collective behaviors were: 1. climate education, 2. civic participation, 3. advocacy, 4. activism. Before the beginning of the study, the participants read some instructions, explaining the 8 behavioral categories in detail (with examples), and how to complete the survey; the instructions were linked at the beginning of every survey.

***Behavior options***

The behaviors listed in the drop-down menus were related to the following. We selected emission-reduction behaviors that are commensurate with a daily report such as EMA (e.g., decisions such as not having children may be too big and long-term to fit a daily behavioral report), and that take into account our student audience (e.g., students may not have decisional power to weatherize their home, as most rent their homes). Additional reported behaviors will only be included for analyses if they fit a behavior in the following list unless the behavior is also relevant for emission reduction according to our sources and judgment.

| **Behavior type** | **Behavioral category** | **Relevant behaviors** |
| --- | --- | --- |
| *Collective* | Civic Participation | - 💰 Donate money to a climate cause/initiative - 📝 Sign and share climate petitions asking for climate policy - 🪧Attend a public climate action such as demonstrations, protests or sit-ins - 🗣️ Speak up about climate action at a public event such as a talk, workshop, class or political event - 🗳️ Vote for pro-climate political candidates |
|  | Advocacy | - 👥🤝Attend the meetings of a climate organization that *negotiates* climate policy with decision-makers - 📃🤝Contribute to a project organized by a climate organization that *negotiates* climate policy with decision-makers - 🧑‍💼🏳️Contribute to a pro-climate program within a political party - 📨🧑‍💼Personally contact decision-makers to ask for climate policy - 🆕🤝Start a new project or organization to *negotiate* climate policy with decision makers |
|  | Activism | - 👥🪧 Attend the meetings of a climate organization that *puts pressure* on decision makers to enact climate policy - 🪧📢 Organize a public climate action or project to *put pressure* on decision makers to enact climate policy - 🏛️ Participate in court actions against major emitters - 🪢 Build networking among activists or activist groups - 🆕🪧 Start a new project or group to *pressure* decision makers to enact climate policy |
|  | Education | - 💬 Talk to people about the climate crisis and the actions they can do - 📱🌡️ Share information on the climate crisisvia social media - 📱🪧 Share information on events organized by climate organizations via social media - 📱🧑‍💼 Share information on pro-climate political candidates/proposals via social media - 🧑‍🏫Organize climate education events/projects |
| *Individual* | Food consumption | - 🥗 Replace meat and dairy with vegan options - 🇳🇱 Only buy local food - 🥦 Only buy seasonal (fresh) food - 🍟🚫 Avoid industrial junk food - 🍽️ Prevent food waste |
|  | Transportation | - 🚲🚋 Replace car use with walking, biking, or public transport - 🚙👥 Opt for shared car rides over individual car use - 🚆 Choose trains, buses, or carpooling instead of flying, if traveling or booking transports for a future trip - 🚙🔌 Rent electric vehicles instead of combustion engine ones - 🚙⏲️ If using a car, reduce the speed to consume less gas |
|  | Energy use | - 🛁 Use less hot water to wash yourself and clean - 🥵Reduce heating/cooling in your house/work/university - 💡🔌 Turn off lights and unplug devices/appliances when not in use - 🍳 Limit cooking gas use - 🌐 Reduce transfer and storage of heavy data (e.g., emails, social media) |
|  | Finances | - 🤷 Avoid buying unnecessary items - 🛍️ Avoid buying new, low-quality items produced far away - 💻🛍 Avoid online shopping, unless in bulk - ♻️🔌 Switch your energy bills to a 100% renewable energy provider - 🏦🍃 Switch your bank/insurance to a bank/insurance that doesn’t fund fossil fuels |

Coding List References:

- Individual behavior:
  - Marchi et al., 2021
  - Brandenstein et al., 2023
  - Dietz et al., 2009
  - Our World in data - Emissions by sector: <https://ourworldindata.org/emissions-by-sector>
- Collective behaviors:
  - Hartley, S. (2025, September 2). *Climate activism: Start here*. The Commons Social Change Library. <https://commonslibrary.org/climate-activism-start-here/>

***EMA Survey, Part 1: Actions performed today***

INDIVIDUAL BEHAVIORS

1. Energy
   i. Did you ______ today? Yes, No

*[where _____ is the action they selected the day before, to conserve energy]*

ii. … *[there will be up to 5 questions like i., depending on how many behaviors were planned the day before for this category]*

2. Food
   a. Did you ______ today? Yes, No
   b. …
3. Transportation
   a. Did you ______ today? Yes, No
   b. …
4. Finances
   a. Did you ______ today? Yes, No
   b. …

COLLECTIVE BEHAVIORS

5. Education
   a. Did you ______ today? Yes, No
   b. …
6. Civic support
   a. Did you ______ today? Yes, No
   b. …
7. Advocacy
   a. Did you ______ today? Yes, No
   b. …
8. Activism
   a. Did you ______ today? Yes, No
   b. …

***EMA Survey, Part 2: Action plan for tomorrow***

INDIVIDUAL BEHAVIORS

1. Energy:

What are you going to do tomorrow to conserve energy? Select up to 5 behaviors from the dropdown menu. [The menu will show multiple answers and the participant can select up to 5]

2. Food:

What are you going to do tomorrow to reduce your diet carbon footprint? Select up to 5 behaviors from the drop-down menu.

[Drop-down menu will show multiple answers, the participant can select up to 5]

3. Transportation:

What are you going to do tomorrow to reduce your transportation carbon footprint? Select up to 5 behaviors from the drop-down menu.

[Drop-down menu will show multiple answers, the participant can select up to 5]

4. Finances:

What are you going to do tomorrow to reduce your finances' carbon footprint? Select up to 5 behaviors from the drop-down menu.

[Drop-down menu will show multiple answers, the participant can select up to 5]

COLLECTIVE BEHAVIORS

1. Education:

What are you going to do tomorrow to educate people around you about climate mitigation? Select up to 5 behaviors from the drop-down menu.

[Drop-down menu will show multiple answers, the participant can select up to 5]

2. Civic support:

What are you going to do tomorrow to participate in civic initiatives for the climate? Select up to 5 behaviors from the drop-down menu.

[Drop-down menu will show multiple answers, the participant can select up to 5]

3. Advocacy:

What are you going to do tomorrow to engage in climate advocacy? Select up to 5 behaviors from the drop-down menu.

[Drop-down menu will show multiple answers, the participant can select up to 5]

4. Activism:

What are you going to do tomorrow to engage in climate activism? Select up to 5 behaviors from the drop-down menu.

[Drop-down menu will show multiple answers, the participant can select up to 5]

**FINAL SURVEY**

This will be like the Baseline Survey, but measuring

- the 12 psychological factors
- Anxiety

**FOLLOW-UP SURVEY**

This will be like the Baseline Survey, but measuring

- the 12 psychological factors
- Anxiety
- Behavioral Recall

**FOCUS GROUP**

(Example schedule)

Participants were instructed to re-read their module diaries before the focus group and have them within reach during the session.

| **Introduction** | **Now I want to ask you some questions about your experience within this study.** |
| --- | --- |
| **1.** | Can you think of any particular element of the intervention (module/activity/format/tutor’s approach) that inspired you to get more involved with climate action? |
| **2.** | Can you think of any particular element of the intervention (module/activity/format/tutor’s attitude) that was not as inspiring or left you feeling unsure about engaging with climate action? |
| **3.** | Which behavioral category (individual or collective) you found particularly easy to engage in? And why? |
| **4.** | Which behavioral category (individual or collective) you found particularly difficult to engage in? And why? |
| **5.** | Was there any topic or aspect of the climate crisis that you would have liked to learn more about? Or that you found redundant? Or any topic that was not there and you would have liked to learn about? |
| **6.** | Do you have general suggestions on how to improve the intervention to make it more empowering for individual / collective climate action? |
| **In-depth: Questions for 1 - 2** | a. What exactly did you think about this module/activity?  b. How exactly did you react/feel?  c. Can you describe why this was so [inspiring/uninspiring]?  d. In what ways did this alter your views on the climate crisis? |
| **In-depth: Questions for 3 - 4** | a. What exactly made it easy/difficult to perform this behavior?  b. Did you plan for it and failed to perform it, or did you simply not have opportunities to engage (and therefore did not plan for it)?  c. For difficult behaviors: what do you think would make it easier for you to engage? |

**INTERVENTION CONTENT TABLE**

**Supplementary Table 1.** *Intervention modules, with the respective psychological factors targeted and example activities used to boost each target factor. Existing interventions or theories related to the activities are referenced.*

| **Module** | **Factors Targeted** | **Short activity description** |
| --- | --- | --- |
| **Module 1:**  **Intro to the climate crisis and climate action.**  On the causes, impacts of the climate crisis, and on the psychological obstacles/incentives of mitigation behavior (3h) | Appraisal | Whole class. Interactive workshop on climate attribution analysis: understand how scientists know that increasing global temperatures lead to extreme weather in a given region  (Nordlund & Garvill, 2003) |
|  | Emotional Engagement (risk, anger, empathy) | In groups: View and discuss an anger consensus message about climate inaction (statistics are presented about a growing number of people being angry about climate inaction)  (Castiglione et al., 2023; [Sabherwal et al., 2021](https://pdf.sciencedirectassets.com/272402/1-s2.0-S0272494421X00047/1-s2.0-S0272494421000931/main.pdf?X-Amz-Security-Token=IQoJb3JpZ2luX2VjEJX%2F%2F%2F%2F%2F%2F%2F%2F%2F%2FwEaCXVzLWVhc3QtMSJIMEYCIQDMcBZEM1wEZa7SdWVWbXmMcezNkWi8d1BFRDSCqNKxWwIhAI31BcTB2vF1gDo8s6bPAr4WW0NGZfThd9rhaRAZ%2Bns8KrwFCO7%2F%2F%2F%2F%2F%2F%2F%2F%2F%2FwEQBRoMMDU5MDAzNTQ2ODY1IgwQBBFyt4taODmPeoMqkAXQ4XYfaTQrUCl9yqtecw%2BgQy0WxTd%2BnhIz2boAyKm9NGCXQs31CBZIbNQxx%2BTGyH1EicXj3DHUQD7b0hIs0pmsQhTFdCzupbp5kDpbWQtg4RviPcR%2FLnJigyftqMj7tf2Wq9S2hY9IhipBEPmQmx4Rh4at8F8oEa7nt61lvbkaqUpBQ2%2Fp5xhwVeJzREF9w4CSl9O9X7ihPRx005%2B78SRE7T9Ig5bI2wA5xoNmpbPD4wVohfEdrLUEZPCzIeM8GXdJ1AuNV%2BRjYCdSj%2BWZRc0H%2BWmFaLo6AklVsKlP6B1fdZvnUuun%2BcHvXWMtNPGD5g9qG7ATWqnzaOruiwsixiE1OeCdAPyw08Sy4DxineV1vHti1TAcEhNa0u2M2BMjocS6iJBpMC4VmwLfIu1ZmIciyacXFVgk5HAo5LoUF0rctW5bYXg7mzTShEyCX3fGRU%2BPyDGj%2Bs7uSYQTrHdNYEMzxOZhWxYDlyxwQA5MW%2FZi%2BSkWW%2BXBHllj4rzCj3uOUNfCJ4Db6%2BPqRghT6fLJ0AbRKy3CqZmaPJkPWPJvzd1lGpSP17Bx%2FGc3Eo%2B7TRVA884gA4lBn6WssS2Wg5LEo2CJzR2wA%2BFMdIV5ile7cv1cuaID6InBsYS%2BkBJ3ujj2g5oztzDWhob1ztAofmA%2B7qSwuC0FvB9Nnph%2BuBsN%2FLGnVxwMk%2FV9dTNgnNOApZltv7aE41gDNlEhJ3q6tRI2Sd71VuLWG4%2BmBLSEW5RTKfmne9GsdWxlkP4sQLtBrq0MPgeKvDUyzSURze7LtCPn21dRx4LJh8odhQ%2BKkvZ6a8OkuyPwjYKhwoT36zDHcMC3PVlC%2BXylVKPuQOtuw6wFfGsr0SxrbgKY0UO4KNvA87eH2zC6kKKrBjqwATOT9WAXjt3FAAnARjTD2WHDolrXc20XhkacCc1%2BVJ3mcA8l%2B0AaQ43b7Lsbp8LVtuOuboICzmQlQDyTMEhwy0I%2BQeHNHymF2BsLaZTWMmcIjQ1iCnhL%2Fp0QY2YeLgy8wqLJXN%2FwQ9XoboWaNqw4skplxz0S5rsgWjb%2BzUYKFE6OYjszG%2FnqVIpSq4x%2FjI9vjS4HvXfyWe2LO5RNNkerbjoBAXWIHCcF7%2FTdbVm55JP9&X-Amz-Algorithm=AWS4-HMAC-SHA256&X-Amz-Date=20231130T140642Z&X-Amz-SignedHeaders=host&X-Amz-Expires=300&X-Amz-Credential=ASIAQ3PHCVTY5TGH44NA%2F20231130%2Fus-east-1%2Fs3%2Faws4_request&X-Amz-Signature=6703e7defa96bd348934157406110fc58545c0ad2b67111de2d66bb4914d1635&hash=28732de9ca5d4051525859183c92a65ff9b37d578e7e3d26a1984057a32a01ac&host=68042c943591013ac2b2430a89b270f6af2c76d8dfd086a07176afe7c76c2c61&pii=S0272494421000931&tid=spdf-bcb59738-36d5-464c-a219-24834093a4fb&sid=11ddc11564b13247116b39e25c3f739ee9d2gxrq); van der Linden 2017) |
|  | Self-identity | Alone + group discussion. Reflect on and discuss your environmental emotions, values and behaviors:   - Values: what are nature and the environment to you? Are they important to your well-being? How and why do you believe they should be preserved? Are humans above or at the same level as the rest of the natural world? Let naturalistic values emerge - Behaviors: how do you act on your values? What do you do / have you done to protect the environment? Think of any example in your life, and share it   ([Lalot, 2019](https://www.sciencedirect.com/science/article/pii/S0272494418303463?casa_token=JY2_5wzfvJMAAAAA:7kyqtbYlhHF2U3LSO0WsEG_ywY4NCHDp9kYbkFRCgER__90CHA7Q7iIGhWGysU1fZ-W7FhVS9A); [Lauren, 2017](https://journals.sagepub.com/doi/full/10.1177/0013916517740408?casa_token=NFWvaNGjyScAAAAA%3AkWVQrdJA5SH8vSn36iPDXHjKnRd7WPsDyvv1qIP4PrDx2xQdvh8pciHa6D-MtQdFlRXOHMewns6X); Mayer & Frantz, 2004) |
|  | Collective-identity | Whole class + group discussion.   - Whole class. Watch a short documentary about famous climate advocates/activists close to the audience (like Fridays for Future or Sunrise movement leaders); emphasizing the bottom-up nature of their work. - In groups. Analyze the advocates/activists’ motivations: why are they taking action? What are their psychological drives? What do you have in common with them (e.g., psychological drives, age, gender, background)? Can you see yourself among them? Would you be their friend? Can you define yourself as an environmentalist?   (Brick & Lai, 2018; Jans, 2021; [Masson & Fritsche, 2014](https://onlinelibrary.wiley.com/doi/full/10.1002/ejsp.2036?casa_token=3Ri5FKYNyI8AAAAA%3AT_jh6RIpBtOJQojjrxGPAYMBzp2vrYr5D33ubUObm37xSiAZAt4j7LWPBvXrTWAEUcIDgiEQd6Bpllw); Pettigrew & Tropp, 2006) |
| **Module 2:**  **Decarbonization**.  On the main greenhouse gasses, their anthropogenic sources, and how to cut them (3h) | Self-Efficacy | In groups.   - Run a small research project on how much emissions would decrease if every person: A. stopped eating meat, B. stopped using cars/planes, C. moved to renewable energy, D. only bought used things. - Brainstorm: how can your individual actions affect others? Develop a communication strategy leveraging a psychological factors of your choice (learned form last time), to convince somebody around you to engage in a climate mitigation behavior that you do. - Reflect and discuss on the IMPACT of collective versus individual actions   (Hamann et al., 2020; Hornsey et al., 2022; Jugert et al., 2016) |
|  | Collective Efficacy | In groups.   - Read and discuss materials (text and videos) about how young adults, through their civic engagement, have contributed to climate mitigation goals in Italy. - Use the software En-ROADS to learn what would happen to global temperature if we abated the emissions from various sectors of society.   Watch documentary on successful climate action, i.e. group of citizens convincing government or institution to enact some decarbonization procedure (e.g., Fridays for Future, Sunrise.org) and discuss together  ([Bieniek-Tobasco et al., 2020](https://journals.sagepub.com/doi/10.1177/1075547020951794); Hamann et al., 2020; Hornsey et al., 2022; Jugert et al., 2016) |
|  | Social Norm | In groups:   - Read and discuss statistics of what other young adults across the world think about climate action, and how they are increasingly taking more individual and collective climate action. - Now that you have a better idea of the most impactful pro-climate behaviors, share all the individual and collective pro-climate behaviors you already do with the rest of your group. Tell others how you started engaging in these behaviors.   (Constantino et al., [2022](https://journals.sagepub.com/doi/full/10.1177/15291006221105279); [Jachimowicz et al., 2018](https://www.nature.com/articles/s41562-018-0434-0); [Nolan 2021](https://www.sciencedirect.com/science/article/pii/S2352250X21000701?casa_token=c63qLjF26AEAAAAA:XrXjwUua4_TqVnjqXVvicmpg5U3NjlAKx8hFZH3yQbwdFqzt-B2y2GIioh_KnzhqpClPW7b-Kg); [Sparkman et al., 2020](https://www.mdpi.com/2071-1050/12/6/2453)) |
| **Module 3:**  **Climate Economics.** Shifting from a linear fossil economy to a circular economy (3h) | Appraisal | In groups. Run a small research project on fossil finance: how rich is the fossil fuel industry? Which major financial institutions are supporting it (e.g. banks, insurance companies)? In which ways are you supporting it with your money?  Ethical banks: via bank.green (https://bank.green/) find how ethical your bank is and whether there is a better bank in your area to move your money to.  (Nordlund & Garvill, 2003) |
|  | Controllability | In groups. Build a personal decarbonization plan to facilitate your own emission reduction. Set SMART goals for reducing your CO2 footprint from food, transports, energy and finances. Build a timeline by using a calendar, and nudging strategies for yourself and include a weekly-tracker so you hold yourself accountable.  In groups. Brainstorm tricks to perform complex pro-climate behaviors. 1) Select easily performed behaviors and reason on what makes them easy. 2) List difficult behaviors (e.g., habit change or civic engagement or activism). 3) Brainstorm similar non-climate-related difficult behaviors you already do in life (e.g., behaviors that require habit-change or exposure to the public). 4) Compare: what makes the difficult behaviors you already do feasible? How to transfer this to pro-climate difficult behaviors?  (Fielding et al., 2008; [Steinmetz et al, 2016](https://psycnet.apa.org/fulltext/2016-52618-007.pdf?auth_token=45eabf07fc40764733c3c7e66050b204e9df4315&returnUrl=https%3A%2F%2Fpsycnet.apa.org%2Frecord%2F2016-52618-007)) |
|  | Implementation intentions | In groups. Explore and discuss a list of collective and individual pro-climate behaviors you would like to implement.  Alone. Build a “where, when, how” plan to implement your personal decarbonization plan, including the specific behaviors you just analyzed in the groups.  In groups. Explore a social media channel advertising activism/advocacy events in your region. Select some events that you would like/it is most feasible for you to attend, based on the place, time and characteristics of the event.  (Gollwitzer & Sheeran, 2006; [Steinmetz et al, 2016](https://psycnet.apa.org/fulltext/2016-52618-007.pdf?auth_token=45eabf07fc40764733c3c7e66050b204e9df4315&returnUrl=https%3A%2F%2Fpsycnet.apa.org%2Frecord%2F2016-52618-007)) |
|  | Cognitive Alternatives | In groups. Imagine picking an institution (a company, school, city) and imagine what it would look like if it was a zero-emission institution. How would energy work? And the food and transportation system? Where would its money be invested? Write a short essay describing the institution  (Wright et al., 2020) |
| **Module 4:**  **System Change**.  On how societal change can spread from local to global (3h) | Trust in Institutional Politics | In groups. Run a research project on political figures in the history of your country who have pushed for/implemented climate mitigation policy, in response to citizen pressure.  ([Feldman et al., 2015](https://journals.sagepub.com/doi/10.1177/1075547015617941)) |
|  | Cognitive Alternatives  + Theory of Change | In groups: imagine how the change in the institution you worked with in module 3 can spread to other similar ones. Imagine you built a campaign to change the institution, and you made it! How could this change escalate and diffuse from that institution to similar ones, up to the global scale?  (Schmitt, 2023; Wright et al., 2020) |
|  | Collective Efficacy | Watch documentary on successful climate action, i.e. group of citizens convincing government or institution to enact some decarbonization procedure (e.g., Fridays for Future, Sunrise.org) and discuss together  ([Bieniek-Tobasco et al., 2020](https://journals.sagepub.com/doi/10.1177/1075547020951794); Hamann et al., 2020; Hornsey 2022; Jugert et al., 2016; Hornsey 2022; [Bieniek-Tobasco et al., 2020](https://journals.sagepub.com/doi/10.1177/1075547020951794)) |
| **Module 5:**  **Civic engagement for climate, part I**.  On how to create effective bottom-up change (3h) | Self Efficacy | Alone: Reflect on your skills: what are you good at? What do you like to do? E.g. speak in public, write, make art, plan, do research, use social media. How can you apply these skills to civic participation for a climate cause? What could be your role in a climate group/organization?  (New) |
|  | Collective Efficacy | In groups. Divide into interest groups around a climate issue (transportation, food, finances, energy) and plan a climate mitigation campaign in a place of your choice (pick among your university, your city, your region). Identify a goal, a decision maker to convince to implement your goal (target), a strategy to convince the target, a timeline, distribute roles and responsibilities within your group, build a monitoring plan. Write a short report of your campaign.  ([Lacerenza et al., 2018](https://pubmed.ncbi.nlm.nih.gov/29792465/); Raue et al., 2013) |
| **Module 6:**  **Civic engagement for climate, part II**.  On effective team work for climate action (3h) | Collective Identity  + Collective Efficacy | In groups.   - Guided brainstorm on team-work: what effective teamwork looks like? How to define roles? How to distribute responsibility? How to lead a group? How to be accountable? - Applied laboratory: continue working on the campaign you started developing in module 5, but now apply the team-work guidelines brainstormed above to your campaign group (get to know each other, define roles, distribute responsibility, define leadership and accountability guidelines…) - Revisit your campaign plan (e.g., tactics, timeline, roles) based on your new team-work guidelines - Group discussion. Do you feel closer to your group now that you are working as a real team? Do you feel you could have an impact if you kept working on this campaign?   ([Lacerenza et al., 2018](https://pubmed.ncbi.nlm.nih.gov/29792465/); Raue et al., 2013) |
|  | Implementation Intentions | In groups. Make an implementation plan for your campaign: when, where and how can you really implement it? E.g. can you do it through a climate organization? Which one? How/when will you contact them to propose your idea?  (Gollwitzer & Sheeran, 2006; [Steinmetz et al., 2016](https://psycnet.apa.org/fulltext/2016-52618-007.pdf?auth_token=45eabf07fc40764733c3c7e66050b204e9df4315&returnUrl=https%3A%2F%2Fpsycnet.apa.org%2Frecord%2F2016-52618-007)) |
|  | Controllability | Alone. Build a “personal civic engagement plan” to  increase your collective pro-climate behaviors. Use a calendar, and nudging strategies for yourself and include a weekly-tracker to hold yourself accountable.  (Fielding et al., 2008; [Steinmetz et al., 2016](https://psycnet.apa.org/fulltext/2016-52618-007.pdf?auth_token=45eabf07fc40764733c3c7e66050b204e9df4315&returnUrl=https%3A%2F%2Fpsycnet.apa.org%2Frecord%2F2016-52618-007)) |
|  | Social Norm | Whole class + in groups:   - Listen to a presentation by members of some local climate organizations (where most members are young adults) to speak about their organization, how they build climate mitigation campaigns and initiatives, their achievements, and ways in which people can join. - Divide into groups, the activists go around the groups to respond to questions and discuss climate engagement with the participants.   (Constantino et al., 2022) |

**INTERVENTION MEASURES**

***Comprehension questions***

There will be 4 comprehension questions for each module. 2 questions will be about the content from the 40-minute presentation, and 2 questions will be about the activities carried out during the module.

Example comprehension question for Module 1:

1) How does the greenhouse effect work?
   a) The greenhouse gasses in the atmosphere retain the infrared radiation emitted by the earth, warming up the bottom layer of the atmosphere and thus the earth surface
   b) The greenhouse gasses in the atmosphere retain the infrared radiation emitted by the sun, warming up the top layer of the atmosphere and thus the earth surface
   c) The infrared radiation emitted by the earth retains the greenhouse gasses in the atmosphere, warming up the earth surface
   d) The infrared radiation emitted by the sun retains the greenhouse gasses in the atmosphere, warming up the earth surface
2) Which kind of extreme weather has increased in the Netherlands due to global warming?
   a) Floods
   b) Draughts
   c) Tornadoes
   d) Both a and b are correct
3) What was one of todays’ *activities* centered around?
   a) your emotions regarding climate change
   b) linear versus circular economy
   c) the carbon market
   d) climate policy
4) What was the other activity about?
   a) find out extreme weather in your region
   b) climatological modeling
   c) fast fashion
   d) consumerism

**SHORT PSYCHOLOGICAL QUESTIONNAIRES**

For each module, this will consist of a section of the baseline psychological questionnaire, including all the psychological factors targeted by the module (e.g., for Module 1, this will include Emotional Engagement and Appraisal questions).

**MODULE DIARY**

**At the end of each module, participants will be prompted to write a short diary page, responding to the following questions:**

1. Write a summary of what happened during today’s module (2 sentences)
2. What did you find motivating, encouraging, empowering about today’s:
   a. contents and activities? (1 sentence)
   b. format (e.g., time spent on the presentation or doing each activity) (1 sentence)
   c. tutor’s attitude (1 sentence)
3. What did you find boring, demotivating, disempowering about today’s contents and activities? (1 sentence)
   a. contents and activities? (1 sentence)
   b. format (e.g., time spent on the presentation or doing each activity) (1 sentence)
   c. tutor’s attitude (1 sentence)
4. How can you use what you learned today to increase your individual and collective pro-climate behaviors? (2 sentences)

**INCENTIVE SYSTEM AND EXCLUSION**

***BASELINE + FINAL***

**Experimental group**

Participants will start with 0 points and can earn up to 134 points. They will earn proportionally to the effort and time spent on the corresponding task, as follows:

- 1 point for every EMA survey completed (x28 days)
- 0.5 points for every correct answer to a module comprehension question (x24 questions)
- 10 points for every meeting they attend (x6 meetings)
- 10 points for participating in the focus group (x1 focus group)
- 3 points for every module short-psychological questionnaire (x6 questionnaires)
- 1 points for every homework they complete (x6 homework)

At the end of the study, all participants reaching a 120 score-threshold will enter a raffle; out of these, 3 randomly selected participants will win a prize worth about 150 euro (which will vary from a cash reward to a gift card, depending on the audience).

**Control group (Study 1):**

Participants will start with 0 points and can earn up to 38 points. They will earn points in the following ways:

- 1 point for every EMA survey completed (x28 days)
- 10 points for participating in the focus group (x1 focus group)

All participants reaching a 30 score-threshold will enter a raffle; out of these, 3 randomly selected participants will win a prize worth about 50 euro (which will vary from a cash reward to a gift card, depending on the audience).

***FOLLOW-UP***

**Experimental and control group**

Participants will start again with 0 points and can earn up to 24 points. They can only win this prize if they were not excluded from the study at the final stage. They will earn points in the following ways:

- 1 point for every EMA survey completed (x14 days)
- 10 points for responding to the follow-up psychological questionnaire

All participants reaching a 15 score-threshold will enter a raffle; out of these, 2 randomly selected participants (per condition) will win a prize worth about 50 euro (which will vary from a cash reward to a gift card, depending on the audience).

**Participants will be informed that they will be excluded from the study if:**

- They fail to complete more than 5 daily surveys (out of 14), in any of the initial and final EMA behavioral tracking stages
- They fail to fill any one of the baseline and final psychological questionnaires
- They fail to attend more than 2 out of 6 intervention meetings (experimental participants only).

**ADDITIONAL FIGURES AND TABLES**

|  | | **Experimental** | | |  | **Control** | | |
| --- | --- | --- | --- | --- | --- | --- | --- | --- |
| **FACTOR** | | **Baseline**  **M (SD)** | **Final**  **M (SD)** | **Follow-up**  **M (SD)** |  | **Baseline**  **M (SD)** | **Final**  **M (SD)** | **Follow-up**  **M (SD)** |
| **Emotional Engagement** | 7.20 (0.54) | | 7.85 (0.56) | 7.54 (0.61) | | 7.17 (0.56) | 7.14 (0.70) | 6.46 (0.77) |
| **Self Efficacy** | 5.43 (1.04) | | 5.81 (0.82) | 6.07 (0.81) | | 5.05 (1.29) | 5.82 (0.83) | 4.18 (1.33) |
| **Collective Efficacy** | 6.57 (0.85) | | 7.71 (0.77) | 7.19 (0.84) | | 6.56 (0.96) | 6.85 (0.98) | 6.21 (1.01) |
| **Theory of Change** | 6.38 (0.78) | | 7.35 (0.92) | 7.28 (0.62) | | 6.14 (1.15) | 6.11 (0.86) | 6.07 (1.12) |
| **Cognitive Alternatives** | 6.46 (1.07) | | 6.88 (1.52) | 6.91 (1.37) | | 5.15 (1.60) | 5.76 (1.45) | 5.9 (1.48) |
| **Controllability** | 6.48 (0.75) | | 6.51 (0.58) | 6.57 (0.67) | | 5.79 (0.83) | 5.98 (0.74) | 5.75 (0.79) |
| **Self Identity** | 6.14 (0.98) | | 6.54 (0.83) | 6.26 (0.81) | | 5.77 (0.82) | 6.04 (0.79) | 6.25 (0.57) |
| **Collective Identity** | 5.08 (1.83) | | 5.86 (1.21) | 5.61 (1.36) | | 4.91 (1.28) | 5.57 (1.47) | 4.69 (0.94) |
| **Appraisal** | 8.86 (0.67) | | 9.44 (0.35) | 9.33 (0.71) | | 9.08 (0.82) | 9.01 (0.65) | 8.65 (1.34) |
| **Trust in Institutional Politics** | 4.49 (0.92) | | 4.86 (0.93) | 4.44 (1.05) | | 4.55 (0.89) | 4.47 (0.79) | 5.01 (0.65) |
| **Implementation Intentions** | 4.66 (1.32) | | 6.00 (1.03) | 5.38 (1.46) | | 4.33 (1.52) | 5.05 (1.50) | 4.63 (0.89) |
| **Social Norm** | 7.01 (0.71) | | 6.94 (0.89) | 7.19 (0.73) | | 7.42 (1.07) | 6.96 (1.17) | 7.00 (1.38) |

**Supplementary Table 2**: *Mean and SD of the psychological factors for each group (experimental and control) at each time point (baseline, final, follow-up), and McDonald’s omega scores for all participants, calculated at baseline.*

*Note.* p < .05 shown in bold.

**Supplementary Figure 1.** *Psychological Factors changes before and after the factor-specific modules.*

| **FACTOR** | **Baseline**  **M (SD)** | **Module 1**  **M (SD)** | **Module 2**  **M (SD)** | **Module 3**  **M (SD)** | **Module 4**  **M (SD)** | **Module 5**  **M (SD)** | **Module 6**  **M (SD)** |
| --- | --- | --- | --- | --- | --- | --- | --- |
| **Emotional Engagement** | 7.23 (0.84) | 7.59 (0.67) |  |  |  |  |  |
| **Self Efficacy** | 5.35 (1.39) |  | 5.65 (1.04) |  |  | 6.31 (1.38) |  |
| **Collective Efficacy** | 6.55 (1.09) |  | 7.21 (1.10) |  | 7.53 (0.83) | 7.52 (1.06) | 7.66 (1.15) |
| **Theory of Change** | 6.39 (1.01) |  |  |  | 7.19 (0.81) |  |  |
| **Cognitive Alternatives** | 6.41 (1.50) |  |  | 6.68 (1.35) | 6.85 (1.55) |  |  |
| **Controllability** | 6.35 (1.19) |  |  | 6.39 (0.81) |  |  |  |
| **Self Identity** | 6.13 (1.27) | 5.64 (0.90) |  |  |  |  |  |
| **Collective Identity** | 5.37 (2.03) | 5.59 (1.69) |  |  |  |  | 6.67 (1.53) |
| **Appraisal** | 8.90 (0.74) | 9.21 (0.58) |  | 9.31 (0.51) |  |  |  |
| **Trust in Institutional Politics** | 4.42 (1.18) |  |  |  | 4.70 (1.14) |  |  |
| **Implementation Intentions Intentions** | 4.68 (1.74) |  |  | 5.64 (1.66) |  |  |  |
| **Social Norm** | 7.01 (0.86) |  | 6.93 (1.31) |  |  |  |  |

*Note*. ‘***’ p < .001, ‘**’ p < .01, ‘*’ p < .05. For each factor, the change over time, over the modules addressing that factor, starting from baseline. Significant effects are marked. The means and standard deviations for each factor at baseline and after each module are reported below.

**Supplementary Table 3:** *Group means and standard deviations of each aggregate behavioral score (Action, Planning, Alignment) at each time point (baseline, final and follow-up).*

|  | | **Experimental** | | | | | |  | | **Control** | | | | |
| --- | --- | --- | --- | --- | --- | --- | --- | --- | --- | --- | --- | --- | --- | --- |
| **FACTOR** | | **Baseline**  **M (SD)** | | **Final**  **M (SD)** | | **Follow-up**  **M (SD)** | |  | | **Baseline**  **M (SD)** | | **Final**  **M (SD)** | | **Follow-up**  **M (SD)** |
| **Collective ACTION** | 0.80 (1.2) | | 1.15 (2.31) | | 0.70 (1.93) | | | | 0.69 (1.69) | | 0.66 (1.59) | | 0.48 (1.49) | |
| **Collective PLANNING** | 0.90 (1.34) | | 1.17 (2.40) | | 0.72 (1.97) | | | | 0.76 (1.73) | | 0.64 (1.59) | | 0.52 (1.73) | |
| **Collective ALIGNMENT** | -0.09 (0.59) | | -0.01 (0.63) | | -0.013 (0.51) | | | | -0.07 (0.35) | | -0.00 (0.50) | | -0.09 (0.50) | |
| **Individual ACTION** | 7.21 (4.51) | | 6.73 (4.46) | | 5.89 (4.10) | | | | 6.61 (4.10) | | 6.99 (4.71) | | 6.21 (4.98) | |
| **Individual PLANNING** | 7.48 (4.77) | | 7.22 (4.92) | | 6.36 (4.32) | | | | 6.93 (4.60) | | 7.22 (4.92) | | 6.08 (5.26) | |
| **Individual ALIGNMENT** | -0.35 (0.91) | | -0.49 (0.93) | | -0.39 (0.99) | |  | | -0.36 (0.76) | | -0.38 (0.79) | | -0.36 0.72) | |

**Supplementary Table 4.** *Descriptive Information about EMA Submissions*

| **Time** | | ***N*** | **Total Daily Submissions** | | | **Mean Daily Submission/**  **Participant** | ***SD*** | | | ***Range*** |
| --- | --- | --- | --- | --- | --- | --- | --- | --- | --- | --- |
| **Baseline** | 58 | | | 960 | 16.6 | | | 3.80 | 5 - 20 | |
| **Final** | 51 | | | 832 | 16.3 | | | 3.93 | 6 - 20 | |
| **Follow-up** | 35 | | | 585 | 16.7 | | | 2.76 | 9 - 20 | |

*Note.* Mean daily submissions (daily surveys submitted) greater than 14 indicate that some participants filled out extra surveys during the EMA buffer week.

**Supplementary Table 5.** *GLMER model statistics of the Time x Condition interactions of all the specific EMA action categories (collective: civic participation, education, advocacy, activism, and individual: transportation, food, energy, finances), compared across time (from baseline to final, and from baseline to follow-up). Note. p < .05 shown in bold.*

**Supplementary Table 6.** *Sensitivity analysis, for Baseline-Final tests*

| **Hypothesis** | ***N*** | **df_num** | **df_den** | **Power** | $\boldsymbol{f}^{\boldsymbol{2}}$ | ***η^2^*** |
| --- | --- | --- | --- | --- | --- | --- |
| 1 | 58 | 1 | 56 | .85 | 0.16 | 0.14 |

| **Hypothesis** | ***N*** | **Design** | **Replicates** | **Power** | ***d*** |
| --- | --- | --- | --- | --- | --- |
| 2 | 51 | 2x2 mixed | 14 | .85 | 0.45 |

| **Hypothesis** | ***N*** | **df_num** | **df_den** | **Power** | $\boldsymbol{f}^{\boldsymbol{2}}$ | ***η^2^*** |
| --- | --- | --- | --- | --- | --- | --- |
| 3 | 51 | 1 | 47 | .85 | 0.19 | 0.16 |

*Note.* N changes from H1 (N = 58) to H2/H3 (N = 51) because N = 7 participants completing the psychological surveys did not complete the minimum required EMA surveys; their data was retained in the H1 analysis and excluded from the H2/H3 analyses. The table indicates that we had power = 0.85 to detect the following effect sizes:$f^{2}$ = 0.16 for H1, d = 0.45 for H2, and $f^{2}$ = 0.19 for H3.

**Supplementary Figure 2:** *Behavioral recall plots*

**Supplementary Figure 3:** *Climate anxiety plot*

*t*

*Note.* There was no significant time x condition

interaction for any of the time comparisons for

Climate Anxiety

**Supplementary Table 7.** *Feedback given by the participants on the six intervention modules.*

| **Module** | **Motivating/**  **interesting** | **Demotivating/**  **boring** | **Helpful for engagement** |
| --- | --- | --- | --- |
| **Module 1:**  **Intro to the climate crisis and climate action.**  On the causes, impacts of the climate crisis | - Inspiring and specific examples of successful activism (23%) - Clear explanation of the impacts and mechanisms of anthropogenic climate change (17%) - Nice group discussions: Learning from each other and experiencing a sense of shared desire to engage (14%) | - Overly familiar concepts about anthropogenic climate change – lack of new information (19%) - Hearing about the extent of the consequences of climate change, ongoing pollution, etc. – hopelessness (11%) | - Concrete examples: Gave hope and a better/more applied understanding of collective action (23%) - Understanding the mechanisms of climate change – feeling the urgency and acquiring tools to educate others (20%) |
| **Module 2:**  **Decarbonization**.  On the main greenhouse gasses, their anthropogenic sources, and how to cut them | - Specific examples of successful activism (29%) - Learning what would happen if various kinds of emissions were drastically lowered using an online software (En Roads; Climate Interactive & MIT Sloan, 2025) (11%) - Researching and sharing the positive results of past activism within groups (11%) - Learning how to create a communication campaign to convince another person to take action (11%) - Concrete suggestions for actions and to take and things to demands in advocacy efforts (7%) | - Reading and discussing papers describing successful collective actions within groups (it was hard to follow / boring) (7%) - Exchange within groups about individual actions the participants take (most people only took individual actions) (7%) | - Emphasis on the importance of collective action as opposed to individual action alone (22%) - Specific examples of past collective action successes (hopeful and instructive) (14%) - Understanding the impacts of large-scale emission reduction (14%) - Advice on what concrete changes to ask for to governments and corporations (SMART Goals, concrete timelines, transition plans, etc.) (11%) - Learning how to communicate with others: useful for motivating others to take action (7%) |
| **Module 3:**  **Climate Economics.** Shifting from a linear fossil economy to a circular economy | - How capitalism and inequality fuel climate change (16%) - Planning how to change an institution’s actions – made change seem feasible (13%)Disproportionate climate impact of a few companies and wealthy individuals (10%) - Concrete examples of cutting emission, like changing bank (6%) - Climate action is possible at all levels—local and global (6%) - Planned economic degrowth (6%) | - Achieving change in the participants’ own institutions seemed unrealistic – difficult to identify decision makers (13%) - Complex, deeply unequal systems make climate action seem difficult (10%) - The (ir)responsibility of large companies (6%) - Too much theory in the first part (6%) | - Addressing how we can transform our economy to protect people and the planet (23%) - Brainstorming sector-specific emission reductions (13%) - Handout with concrete recommendations for climate action (10%) |
| **Module 4:**  **System Change**.  On how societal change can spread from local to global | - Seeing the successes of various bottom-up movements; how small movements can grow into large-scale social transformations (52%) - Researching political actors that have made positive contributions to combating climate change (13%) |  | - Seeing how apparently small collective actions can lead to global change; success stories (73%) |
| **Module 5:**  **Civic engagement for climate,**  **part I**.  On how to create effective bottom-up change | - Planning a real campaign to influence decision makers (28%) - Learning that activists do research and write reports, and do not only demonstrate in the street (10%) | - Setting up the campaign; groupwork was difficult (7%) | - Developed a concrete idea of how to set up an activist campaign (46%) - Learning about activism opportunities and how everyone can take part; all skills are needed (21%) - Identifying targets (decision makers) and those who influence them seemed to make change more structured and realistic (11%) - Advice for doing good research before building a campaign (11%) |
| **Module 6:**  **Civic engagement for climate,**  **part II**.  On effective team work for climate action | - Learning about activism opportunities and how everyone can take part; all skills are needed (28%) - Getting specific information about real activist organizations (e.g. how they operate, their active campaigns) (25%) - Presentations by guests from various activist groups (17%) - Learning that the activists met successfully made changes in the past (14%) - Joining an organization can be low-effort: you do not to be involved 100% of your time (7%) |  | - Specific information on organizations, how to join, what tasks one can carry out, etc. (42%) - Teamwork workshop: it was possible to identify with particular activist roles (14%) - Talking to guests from various activist groups (7%) |

*Note.* Percentages were calculated based on how many participants mentioned a given concept, out of all the participants present that day.

**Supplementary Table 8**. *Summary of the results from the final focus group.*

| **Prompt** | **Answers** |
| --- | --- |
| **What elements of the intervention inspired you to engage more with climate action?** | • Learning about campaigns and collective action felt empowering • Practical planning tools and examples of successful climate action campaigns were motivating • Community and peer support from other participants increased engagement  • Joining a social media channel advertising real-world activist events in Amsterdam |
| **What elements were less inspiring or left you unsure?** | • Some theoretical activities without real outcomes (like emotional/value sharing) • Campaign building was sometimes discouraging because too complex • Hard for international students to engage collectively due to legal risks and barriers |
| **Which behaviors were easiest to carry out?** | • Simple individual actions (diet, small individual habits) • Educational activities |
| **Which behaviors were most difficult to perform?** | • Advocacy and activism (require planning and time) • Contacting decision-makers • Joining organizations |
| **Which behaviors changed the most for you after the intervention?** | • Increase in collective action (joining groups, protests) • More plant-based/local food • Greater knowledge and engagement with climate content online • Educating peers about climate action |
| **What changes came from realizing that the impact of collective action can be greater than the impact of individual actions (alone)?** | • More motivation to engage in collective action • Acceptance that individual lifestyle does not need to be perfect • Shift in mindset and greater support of systemic change |
| **What made behaviors easy or difficult to implement?** | • Difficult: lack of time, cost, or geographic distance from collective action sites  • Easy: when actions align with habits or are done with peers |
| **Which topics should have been covered more/less?** | • Basic information about climate change were useful for some, redundant for others • Desire for more political group discussions • Real campaigns preferred over simulated ones: desire to really implement campaigns that were built by the groups • Interest in learning more about the positive impacts of climate action (health, well-being, professional opportunities) |
| **General suggestions to improve workshops?** | • Less theory, more interactive activities • More activities that have real-world impact (e.g. implementing campaigns) • Let participants exchange contacts to stay in touch and engage in climate action in the future |

*Note.* The focus group was carried out online via Zoom, lasted 1.5 hours, and included five participants.

**DEVIATIONS FROM PRE-REGISTERED STUDY**

All deviations from the pre-registered study are explained here, and summarized in Supplementary Table 9.

***Design***

We started recruitment directly after ethics approval and two months before the program, via physical flyering on campus near departments teaching environmental, social, and natural sciences, targeted emails to students taking environmental courses, and using social media. Yet, we could not recruit enough participants for the control group who were also attending environment-related courses. Therefore, after recruiting about 15 control participants (about 40% of the control group), we extended the recruitment for the control group to anybody who wanted to participate to complete the questionnaires in the study. This led to a mixed active/passive control group.

***Exclusion Criteria***

Given the low sample size, we aimed to retain as much data as possible and therefore revised the minimum survey completion threshold, setting five surveys as the minimum required to capture participants’ behaviors across the intervention stages. We considered five daily surveys the smallest number that would still provide a sufficient time window to meaningfully assess participants’ behavioral change across time periods.

**Supplementary Table 9.** *Deviations from registered analyses, with the rationale for each change.*

| **SECTION** | **ORIGINAL PLAN** | **DEVIATION** | **REASON** |
| --- | --- | --- | --- |
| **Design** | The control group is recruited only among students enrolled in in-person courses related to the environment/climate happening at the same time as the intervention, to complete questionnaires on their emotions/beliefs/ behaviors related to the climate crisis. | Only a portion of the control group attended in-person courses related to the environment/climate (about 40%). | We could not recruit enough participants for the control group from environment-related courses, so we extended their recruitment to anybody who wanted to participate to complete the questionnaires in the study. |
| **Exclusion Criteria** | Participants will be excluded from the study for the following reasons: A. they complete fewer than nine daily EMA surveys out of 14 (i.e. they miss more than five surveys). | Participants will be excluded from the study for the following reasons: A. they complete fewer than five daily EMA surveys out of 14 (i.e. they miss more than 9 surveys). | Given the low sample size, we aimed to retain as much data as possible and therefore revised the minimum survey completion threshold, setting five surveys as the minimum required to capture participants’ behaviors across the intervention stages. |
